# Field-scale monitoring of nitrate leaching in agriculture: assessment of three methods

**DOI:** 10.1007/s10661-021-09605-x

**Published:** 2021-12-06

**Authors:** Hannah Wey, Daniel Hunkeler, Wolf-Anno Bischoff, Else K. Bünemann

**Affiliations:** 1 Centre d’hydrogéologie et Géothermie (CHYN), University of Neuchâtel, Neuchâtel, Switzerland; 2 TerrAquat, Nürtingen, Germany; 3 Research Institute of Organic Agriculture (FiBL), Frick, Switzerland

**Keywords:** Nitrate, Leaching, Agriculture, Mitigation, Monitoring, Techniques, Field-scale, Suction cups, Nmin soil sampling, Self-integrating accumulators, Passive sampler

## Abstract

Deterioration of groundwater quality due to nitrate loss from intensive agricultural systems can only be mitigated if methods for in-situ monitoring of nitrate leaching under active farmers’ fields are available. In this study, three methods were used in parallel to evaluate their spatial and temporal differences, namely ion-exchange resin-based Self-Integrating Accumulators (SIA), soil coring for extraction of mineral N (Nmin) from 0 to 90 cm in Mid-October (pre-winter) and Mid-February (post-winter), and Suction Cups (SCs) complemented by a HYDRUS 1D model. The monitoring, conducted from 2017 to 2020 in the Gäu Valley in the Swiss Central Plateau, covered four agricultural fields. The crop rotations included grass-clover leys, canola, silage maize and winter cereals. The monthly resolution of SC samples allowed identifying a seasonal pattern, with a nitrate concentration build-up during autumn and peaks in winter, caused by elevated water percolation to deeper soil layers in this period. Using simulated water percolation values, SC concentrations were converted into fluxes. SCs sampled 30% less N-losses on average compared to SIA, which collect also the wide macropore and preferential flows. The difference between Nmin content in autumn and spring was greater than nitrate leaching measured with either SIA or SCs. This observation indicates that autumn Nmin was depleted not only by leaching but also by plant and microbial N uptake and gaseous losses. The positive correlation between autumn Nmin content and leaching fluxes determined by either SCs or SIA suggests autumn Nmin as a useful relative but not absolute indicator for nitrate leaching. In conclusion, all three monitoring techniques are suited to indicate N leaching but represent different transport and cycling processes and vary in spatio-temporal resolution. The choice of monitoring method mainly depends (1) on the project’s goals and financial budget and (2) on the soil conditions. Long-term data, and especially the combination of methods, increase process understanding and generate knowledge beyond a pure methodological comparison.

**Supplementary Information:**

The online version contains supplementary material available at 10.1007/s10661-021-09605-x.

## Introduction

Problems with deteriorating water quality have emerged worldwide during recent decades. This issue is partly linked to intense agriculture that plays a crucial role in environmental pollution (Rockstrom et al., 2009) and, specifically, in the degradation of groundwater quality (Böhlke, 2002). One critical compound in this context is nitrate (NO_3_^−^) originating from crop nitrogen (N) fertilisation. N is added to soil as it is the main limiting factor for crop growth in agricultural production (Knittel et al., 2012). However, excess quantities of N are leached in the form of NO_3_^−^ due to its negative charge and subsequently transported from the soil compartment through the vadose zone into the aquifers (Cameron et al., 2013). Consequently, NO_3_^−^ is the most common pollutant of aquifers, resulting in failure to meet quality criteria in 18% of the European groundwater body areas (European Environment Agency, 2018). A certain loss of N is inevitable in agricultural systems (Adesemoye et al., 2008; Jabloun et al., 2015). More specifically, the NO_3_^−^ leaching rate increases with fertiliser input (Cameron et al., 2013; Steinshamn et al., 2004). Several studies specified that this nitrate leaching responds exponentially rather than linearly to the fertiliser load (Wang et al., 2019) or the N surplus (Zhao et al., 2016). Besides the N application rate, additional leaching factors in agriculture are crop rotation, field management including ploughing activities, fertiliser type and timing of application, irrigation, as well as soil type and climatic conditions (Cameron et al., 2013).

To protect groundwater and drinking water quality, nutrient management in agricultural systems gained prominence since the early 1990s (EU Commission, 1991). Several governments on national and regional levels have implemented nitrate abatement strategies in vulnerable catchments, ideally in close collaboration with farmers. These mitigation programs need close and case-specific monitoring to guarantee their effectiveness and efficiency. Typically, groundwater monitoring includes measuring the NO_3_^−^ concentration in drinking water pumping stations, piezometers, wells, drainage pipes, or natural springs. The monitoring enables the identification of long-term trends in nitrate concentration, observations of the impact of the regional mitigation strategy, and data acquisition for comparison of nitrate concentrations to regulatory limits. However, the spatial and temporal resolution of such groundwater monitoring is low. This fact leads to several drawbacks for evaluating nitrate mitigation strategies realised on specific fields (Singh et al., 2017). First, many fields and their individual nitrate remediation strategies are spatially integrated into one single measurement. It is thus only possible to see the mixed effect of all fields and all mitigation measures. Second, as there is a flow path distance between the entry and the outlet of the system, i.e., a given field and the monitoring well, there is a lag of months up to several years between activity on the surface and a visible effect at the monitoring site (Böhlke, 2002; Vero et al., 2018; Wang et al., 2013). This delay needs to be considered in all stages of remediation, e.g., developing and assessing suitable policy and adjusting an existing nitrate mitigation strategy.

Results obtained at groundwater monitoring points are always subject to a multitude of influences. It is thus effectively impossible to trace a specific signal like a “hot spot” or a “hot moment” in NO_3_^−^ leaching with monitoring in the pumping well or spring capture zone only (McClain et al., 2003, Gabriel et al., 2016), or to draw conclusions about the effect of a nitrate remediation strategy applied on a single field. Such large-scale monitoring alone may thus be insufficient to understand the behaviour of nitrate in an agricultural system. To make rational decisions, specific data is often required on the field or sub-field scale, i.e. the scale at which farmers act.

Several techniques are known for soil and vadose zone monitoring at or beneath individual agricultural fields, but no standard method has been defined. Lysimeters can be used to develop an understanding of processes. With these installations consisting of a large vessel filled with a disturbed or undisturbed soil monolith from a field, water flow and solute transport can be investigated (Abdou & Flury, 2004). However, for monitoring the in-situ processes and the heterogeneity in the field, other instruments are needed.

Suction cups (SCs) are versatile and can easily be installed directly in the field. A continuous suction is applied to the tubing system to transport the water from soil pores to the collection unit. In general, SCs allow for continuous pore water sampling in the soil under a specific field (Barbee & Brown, 1986; Grossmann & Udluft, 1991). However, SCs have been criticised for only sampling the soil matrix (Barbee & Brown, 1986; Grossmann & Udluft, 1991; Webster et al., 1993; Fares et al., 2009; Wang et al., 2012, Singh et al., 2017).

In contrast, passive sampler methods based on ion exchange resins (Skogley, 1992) were able to sample bromide transport in macropores under unsaturated flow conditions (Li et al., 1993; Yang & Skogley, 1992). In this method, nutrients are adsorbed to the resin from the percolating soil water until the device is retrieved. The resin is subsequently analysed in the laboratory by desorption. The method is suited for monitoring over an extended period, whereas frequent sampling with subsequent temporal aggregation becomes redundant. The result is related to a specific area and can be scaled up to a time-integrated leaching flux per hectare. Bischoff (2007) developed and validated a specific methodology, resulting in a device called Self-Integrating Accumulator (SIA).

A completely different approach based on soil sampling and extraction of mineral N (N-NO_3_^−^ + N-NO_2_^−^ + N-NH_4_^+^) is often used for nutrient monitoring on the field level. The Nmin value indicates how much plant-available N is currently stored in the soil (Wendland et al., 2018). For soil samples collected in spring, Nmin values are widely used to calculate or adjust the fertiliser level in the upcoming season. In autumn, however, the Nmin value describes the amount of mineral N that was not incorporated into the plant or microbial biomass during the growing season (Klages et al., 2018) and thus is prone to relocation into deeper soil layers (Wendland et al., 2018). Therefore, this Nmin value is regarded as an indicator for the N loss potential during the winter months (Haberle et al., 2009), when leaching is generally higher due to higher precipitation, less evaporation, and limited plant growth and water uptake. In the German federal state of Baden-Württemberg, the direct payments for each farmer even depend on the autumn Nmin value (Umweltministerium Baden-Württemberg, 2001).

The examples described above show that several monitoring systems for nitrate leaching from agriculture are available. All these methods allow measurement in fields under active cultivation, but they differ regarding spatial and temporal resolution as well as workload and financial expenditure. While a few qualitative reviews and partial comparisons exist (Webster et al., 1993; Ramos & Kucke, 2001; Anger, 2002; Fares et al., 2009; Wang et al., 2012, Singh et al., 2017), no systematic comparison has so far been made with a data set acquired in a single field study. Furthermore, a complete compilation and comparison of advantages and disadvantages for nitrate monitoring at field-scale are needed.

The study aimed to compare the spatial and temporal resolution of the chosen monitoring methods and evaluate advantages and disadvantages in the installation, maintenance, and costs to suggest a suitable technique for efficient and effective nitrate groundwater monitoring. Thus, the focus is on a methodological comparison rather than discussing the reasons for differences in leaching itself. The methods selected for this study were suction cups (SCs), Self-Integrating Accumulators (SIA), and Nmin soil coring (Nmin). With this research approach, the nitrate leaching on four agricultural fields was quantified, including current management practices as well as implemented leaching mitigation measures.

## Methodology

### Study site

This study was conducted on four agricultural fields (H1, H2, H3, H4) in the Gäu Valley in the Swiss Central Plateau. The region is characterised by intense agricultural production with silage maize, winter cereals (wheat, barley, and spelt), canola, and pasture (mostly grass-clover leys) as primary crops in the rotation. Irrigation is currently not used for these crops. Fodder is used for local milk and meat production. The crops are fertilised with mineral fertilisers, liquid manure, compost and digestates in amounts following the national recommendations (Richner et al., 2017).

The terrain is flat, with the Jura Mountains bordering the region in the North and the Mittelgäu hill chain in the South. The underlying aquifer used for drinking water production consists of large alluvial terraces of gravel, deposited after the Aare glacier retreat during the Würm Ice Age (Pasquier, 1986; Swisstopo, 2020). In the study area, the aquifer has a thickness of 40–60 m with a water table at 6–10 m below ground (Hunkeler et al., 2015). The predominant soil type is classified as Cambisol (IUSS Working Group WRB, 2015).

The nitrate concentration in the closest drinking water well (in 0.1 to 6.1 km distance to the fields) has been monitored for almost 30 years (Kantonales Amt für Umwelt (AfU) Solothurn, 2020). Values exceeding the legal target concentration (25 mg NO_3_^−^ L^−1^) and almost reaching the legal limit for drinking water (40 mg NO_3_^−^ L^−1^) continue to occur in several pumping stations in the region, even though nitrate mitigation measures in the form of voluntary contracts with farmers have been implemented since the year 2000. These contracts include the partial transformation of agricultural land into extensive grassland and regulations regarding soil coverage in winter, crop rotation, sowing date, and tillage. However, these measures’ impact is not visible in the pumping stations, which may be due to the long lag time in the aquifer or to the potential ineffectiveness of the implemented measures.

The annual mean temperature (1981–2010) is 9.0 °C, and the yearly precipitation is 1129 mm. However, during the years of this study (2017–2020), the temperatures were above average. Two winter storms accompanied by heavy precipitation happened at the beginning of 2018. February 2018 was a relatively cold month with a mean temperature of − 0.5 °C. The summer periods of 2018 and 2019 were both characterised by dry periods. More precisely, the summer months of 2018 were abnormally dry, and in June/July 2019, two heatwaves crossed the country. A stable high-pressure weather system resulted in an abnormally dry and warm period in April 2020.

### General experimental design

The monitoring activities took place from October 2017 to July 2020. Four farmer-managed fields (H1, H2, H3, H4) within a distance of 6 km were selected. H2 and H3 are neighbouring fields and managed by the same farmer. The first selection criteria concerned soil properties, namely no hydromorphic conditions and low stone content in the subsoil. Second, every crop (grass-clover leys, maize, cereal, and canola) was to be present at least twice during the study period. The third condition was the willingness of the farmer to participate in the research project.

Of the three methods, SIA and Nmin were tested in each field, while SCs were only installed in H1, H2 and H4 (Table 1).

### Field properties

The texture of the soils was silty loam (Table 2). Only H1 had stones throughout the soil profile, with an estimated volumetric stone content of 4–9%. The soil bulk density, measured with cylinders (⌀ = 5 cm), varied between 1.55 and 1.78 g cm^−3^. The pH was acidic. The soil organic carbon (Corg) contents in the upper soil layer (0–30 cm) are 23 g kg^–1^ in H1 and 13–14 g kg^−1^ in the other fields.

**Table 1:**
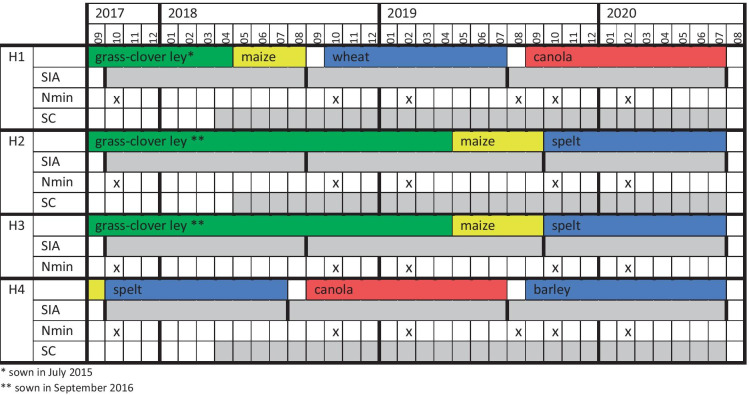
Temporal overview of the investigation period, the crop rotation and sampling frequency on the four experimental fields. The sampling methods include Self-Integrating Accumulators (SIA), Suction Cups (SC), and Nmin Soil Coring (Nmin). Grey shading of a box indicates the temporal integration of a given sample. In contrast, a cross represents a snapshot

### Fertilisation and nitrate leaching mitigation strategies

Until 2019, each field was managed and fertilised as one entity. For the cropping periods 2019 and 2020, each field was divided into three strips to test different nitrate leaching mitigation strategies. Here, we use the values obtained in the different strips to illustrate differences between methods rather than to evaluate the mitigation measures.

The nitrate leaching mitigation strategies on each of the three strips per field concerned the fertilisation of the crops (Table 3). On the first strip, the farmer continued the usual fertilisation (N). On the second strip (M1), the farmer was asked to reduce fertilisation to the recommended level (H2, H3, H4) or to realise split fertilisation (H1). On the third strip (M2), an alternative fertiliser type was used (H4), or the fertiliser amount was further reduced (H1, H2, H3). Due to farm management issues, however, the realised applications did not always fully correspond to the foreseen fertiliser plans.

For the calculation of total N input via organic fertiliser, N concentrations were available either from a manure sample taken by the farmer at the application, from the purchase documentation, or replaced by standard values (Richner et al., 2017), assuming a dilution factor of 1:1 for liquid manure. Total N concentration (Kjeldahl) rather than plant-available N was taken into account.

### Monitoring techniques

The three monitoring techniques used in this study — the Self-Integrating Accumulators (SIA), Nmin soil cores (Nmin), and suction cups (SCs) — differ in the resulting unit (Table 4). While the outcome of the SIA method is a time-integrated flux, i.e. the leached amount of N per area and period, the result of the SC system is a time-averaged concentration. These methods contrast with Nmin soil coring, which gives a snapshot of the soil’s Nmin content at a specific time. Sampling frequency in this study ranged from yearly (SIA) to monthly (SCs), while Nmin samples were taken twice a year (October and February, i.e. pre- and post-winter).

**Table 2:** Soil characteristics of the four experimental fields. Numbers are given for the three horizons of 0–30 cm, 30–60 cm, and 60–90 cm depth

**Field**	**Texture class **^**2**^	**Stones [vol.%]**	**Clay** **[%]**	**Silt** **[%]**	**Sand** **[%]**	**Bulk density** **[g cm**^**−3**^**]**	**pH** **[ −] **^**4**^	**Corg** **g kg**^**−1 5**^
H1	Silty loam /loam	4/4/9	17/11/11	57/51/55	25/38/34	NA ^3^	6.5/6.4/6.6	23/19/10
H2/H3 ^1^	Silty loam/loam	0/0/0	11/10/10	54/53/61	36/37/29	1.68/1.76/1.78	6.1/5.9/5.9	13/6/4
H4	Silty loam	0/0/0	12/12/14	65/66/71	23/21/16	1.55/1.65/1.66	6.3/5.9/5.9	14/6/5

^1^ For H2 and H3, the soil properties were determined jointly since the fields are adjacent

^2^ The texture was determined with Laser Diffractometry, including ultrasound treatment

^3^ In H1, it was not possible to take cylinder samples due to stones

^4^ pH was measured in 0.01 M CaCl_2_ in ratio 1:2.5 W/V

^5^ The Corg was determined from the difference of Ctot and carbonate by direct combustion in a CN Analyser (Vario Max Cube C/N Analysator)

**Fig. 1:**
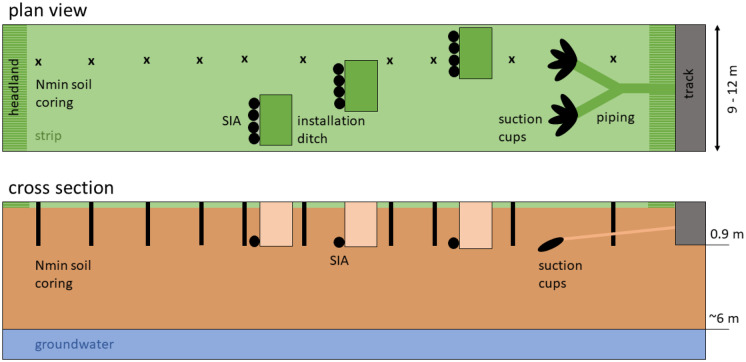
Overview of the installed instruments for monitoring of nitrate leaching with three techniques in parallel

The techniques also differed regarding spatial resolution (Fig. 1). While Nmin soil coring was evenly distributed along a straight trajectory in the entire strip, leaving out the potentially compacted headland used for turning tractors, SIA devices were installed on a diagonal line across the field. Due to physical restrictions in vacuum transport in the piping system, SCs were installed close to the field border right after the headland.

#### SIA measurements

The SIA is a patented passive sampler method developed by the German company TerrAquat (Bischoff, 2007). Field installation, extraction and analysis were performed according to the guidelines, in cooperation and under the guidance of TerrAquat. A single device consists of a plastic cylinder (⌀ = h = 10 cm) filled with sand held by a mesh at the bottom of the instrument. This moistened sand mixture is of predefined hydraulic conductivity and contains an adsorbing resin. The water penetrating the soil by convection flows vertically through the passive sampler. Nitrate is adsorbed to the resin and immobilised.

Three soil pits with four devices each were excavated diagonally across each strip. This approach was already chosen in the first period, when no mitigation measures were in place, aiming at capturing soil heterogeneity (Fig. 1). The instruments were installed under the root zone in 80–100 cm depth inside side tunnels. The devices were thus located under undisturbed soil to maintain the pore structures essential for water flow (Bischoff, 2007).

After recovery, the devices were brought to the laboratory. A subsample of the resin-sand mixture of 15 g was extracted for 30 min with 0.1 l of 1 M NaCl, desorbing the nitrate from the passive sampler resin (Bischoff, 2007). The nitrate concentration was measured via colourimetry using a “Smartchem 450 Discrete Analyser” calibrated for saline solutions. The concentration was transformed into a flux with the following formula:

$$\begin{aligned}&\mathbf N\boldsymbol\;\mathbf f\mathbf l\mathbf u\mathbf x\left[\mathbf k\mathbf g\boldsymbol\;\mathbf N\boldsymbol\;{\mathbf h\mathbf a}^{-1}\right]\\&\quad=\mathbf c\boldsymbol\;\boldsymbol\ast\boldsymbol\;\mathbf v\boldsymbol\;\boldsymbol\ast\boldsymbol\;{\mathbf m}_{\mathbf s\mathbf a\mathbf m\mathbf p\mathbf l\mathbf e}\quad\\&\quad\;\;\;\;{\boldsymbol\;\boldsymbol\ast\boldsymbol\;\mathbf m}_{\mathbf s\mathbf u\mathbf b\mathbf s\mathbf a\mathbf m\mathbf p\mathbf l\mathbf e}^{-1}{\boldsymbol\;\boldsymbol\ast\boldsymbol\;\mathbf r}^{-2}\boldsymbol\;\boldsymbol\ast\boldsymbol\;\mathbf\pi^{-1}{\boldsymbol\;\boldsymbol\ast\boldsymbol\;10}^{-2}\end{aligned}$$

with c being the measured nitrate concentration of the extraction solution [mg N L^−1^], v the volume of the extracting solution [0.1 L], m_sample_ the sand-mixture weight [g], m_subsample_ the sand weight of the subsample [15 g], and r the radius of the SIA device [0.05 m].

The devices were recovered and replaced using the same side tunnels after harvest but before sowing the next crop to limit crop damage in the field, thus in summer (after cereals and canola) or in autumn (after maize and grass-clover ley). In the case of continued multi-annual grass-clover leys, SIAs were exchanged on an annual basis, with the change taking place in autumn. When maize was sown in May after a grass-clover ley, devices were also changed in previous autumn. This approach results in an integrated measurement of a period of 10–13 months (Table 1). Since leaching during summer months contributes very little to the annual loads, SIA results are shown as approximate annual fluxes in kg N ha^−1^.

#### Nmin measurements

A soil sampling campaign was carried out twice a year, namely in February and October (Table 1). This way, the Nmin concentration at the beginning and end of each vegetation period was measured. Samples from February 2018 are missing, as the soil was too wet for sampling before the first fertiliser application.

Ten single samples per strip were taken along at least one trajectory with constant distances (13–20 m, depending on field length) between the subsamples to capture soil heterogeneity (Fig. 1). The samples were taken with an automated sampler down to 90 cm depth, divided into three horizons of 0–30 cm, 30–60 cm and 60–90 cm. Subsequently, the single samples of a given layer were mixed to create one composite sample per field and horizon, frozen, and later analysed in the laboratory (Agroscope, 1996). The steps included a homogenisation using a 4 mm sieve or, where clay content was too high, an 8 mm sieve. 150 g of moist soil was extracted with 600 ml of 0.01 M CaCl_2_ solution for 60 min. The solution was then filtered, frozen, and analysed as described above with a “Smartchem 450 Discrete Analyser” for nitrate, nitrite, and ammonium and, as the sum of it, Nmin.

Simultaneously, 100 g of each sample was dried in the oven at 120 °C for 24 h to determine the gravimetric water content. The following formula allowed transforming the Nmin concentration to a Nmin content per hectare:

$$\begin{aligned}&\mathbf N\mathbf m\mathbf i\mathbf n\boldsymbol\;\mathbf c\mathbf o\mathbf n\mathbf t\mathbf e\mathbf n\mathbf t\left[\mathbf k\mathbf g\boldsymbol\;\mathbf N\boldsymbol\;{\mathbf h\mathbf a}^{-1}\right]\\&\quad=\left[{\mathbf c}_{\mathbf k\mathbf o\mathbf r\mathbf r}\boldsymbol\;\boldsymbol\ast\boldsymbol\;\left({\mathbf v}_{\mathbf s\mathbf o\mathbf l\mathbf u\mathbf t\mathbf i\mathbf o\mathbf n}+{\mathbf v}_{\mathbf w\mathbf a\mathbf t\mathbf e\mathbf r}\right)\boldsymbol\;\boldsymbol\ast\boldsymbol\;\mathbf m_{\mathbf d\mathbf r\mathbf y\mathbf s\mathbf o\mathbf i\mathbf l}^{-1}\boldsymbol\ast10^{-6}\right]\;\\ &\qquad\boldsymbol\ast\boldsymbol\;\left[\mathbf l\boldsymbol\;\boldsymbol\ast\boldsymbol\;\mathbf B\mathbf D\boldsymbol\;\boldsymbol\ast\boldsymbol\;\mathbf S\mathbf t\boldsymbol\;\boldsymbol\ast\boldsymbol\;10^5\right]\end{aligned}$$

with c_korr_ [mg N L^−1^] being the measured Nmin concentration minus the Nmin background concentration in the solvent, v_solution_ the volume of the CaCl_2_ solution [0.6 L], v_water_ the gravimetric water content of the sample [L], m_dry soil_ the mass of the dried soil sample [kg], l the length of the soil core [30 cm], BD the bulk density of the soil [g cm^−3^] and St the stone factor [-] calculated by 1-(stones [vol.%]/100) (Table 2).

The difference between Nmin content in 0–90 cm depth in spring and autumn, in the following annotated as ∆Nmin, was calculated to estimate the Nmin loss during the winter months. With the presented dataset, the calculation was possible for winters 2018/2019 and 2019/2020, with 24 pairs of autumn and spring Nmin data being collected.

#### Suction cup measurements

Suction cups (SIC20 from UMS Meter, ceramic cup head composed of silica carbide, pore size 2 µm (UMS GmbH, 2010) were installed for soil pore water sampling. Per strip, eight suction cups were installed at a distance of 8 m to the field border (Fig. 1). This way, border effects in the headland were omitted, and small-scale soil heterogeneity was represented. SCs and the related tubing system were buried at a minimal depth of 50 cm to allow all agricultural management practices, including tillage, without the research installation being an obstacle for machinery operations (Ramos & Kucke, 2001), and without destroying any material. Thus, the shaft of the SCs was buried below the level of cultivation (Talbot, 2016). The instruments were installed in the walls of excavated pits in previously drilled holes (30° angle to soil surface). This small angle prevents preferential flow along with the instruments (Fares et al., 2009). A body length of 1 m was chosen to position the ceramic cup under undisturbed soil. The drilling holes were filled with a native soil suspension before inserting the cup to guarantee direct contact with soil (Hendrickx et al., 2002, Fares et al., 2009, Singh et al., 2017). The bottles were attached to two batteries and a pump holding a continuous and constant vacuum (− 200 hPa compared to atmospheric pressure) (Hendrickx et al., 2002).

The suction cup’s ceramic tip finally lay approximately in a total depth of 1.20 m, in other words, below the root zone. Water entering the SCs is thought to be “lost” from the soil compartment and would finally reach the groundwater table, as the plant roots cannot take it up anymore and transport it back to the surface.

The water samples were automatically transported to bottles arranged in a concrete chamber at the border of the field, where the vacuum pump was connected. After the installation in autumn 2017, monthly samples were taken between April 2018 and July 2020 (Table 1). All samples were stored frozen without previous acidification, then filtered (“simplepure” syringe filter, 0.45 µm), and analysed for nitrate with ion chromatography (anion analysis with ThermoScientific ICP-1600).

Finally, the SC nitrate leaching flux was calculated by multiplying the NO_3_^−^ concentration in the SC samples with the simulated leaching volume during the same period (van der Laan et al., 2010, Singh et al., 2017). The simulated water flux was also used for calculations in H1, even though the model had not been calibrated for this soil explicitly. The SCs were installed in autumn 2017, but sampling started only in spring 2018. In H1, H2, and H4, it was thus impossible to calculate a SC leaching flux for the entire period 2017/2018. In H3, where no SCs were installed, no information on leaching fluxes is available.

### Water flux model

A soil model is useful for better understanding subsurface hydrological processes and for numerical transformation and comparison of results (van der Laan et al., 2010). With HYDRUS 1D, the water content and water flow were calculated using a one-dimensional, finite element, and single porosity model proposed by van Genuchten–Mualem (Šimůnek et al., 2013). This approach of soil water transport is based on Richard’s equation that is elucidated elsewhere (e.g. Doltra and Muñoz (2010)).

The 150 cm deep soil profile was split into two regions (0–30 cm and 30–150 cm); thus, the horizon influenced by ploughing was distinguished from the remainder. The spatial discretisation (Δz = 1 cm) was uniformly distributed over the soil profile. The model’s total period was from September 2017 to December 2020, but only data from January 2018 onwards are shown to account for model initialisation time. The initial time step was Δt = 0.0005 d, with time steps being limited between 10^–5^ and 10^–3^ d. No hysteresis was allowed in the model.

The input variables were daily precipitation and evapotranspiration (both obtained by MeteoSuisse). The evapotranspiration had been derived with the FAO-56 method, and no separation into evaporation and transpiration was simulated in HYDRUS 1D (e.g. by FAO crop coefficients or the measured Leaf Area Index) due to its complexity (Šimůnek et al., 2008). The default initial water content was set at WC = 0.2 in the entire profile. Free drainage was selected as the lower boundary condition, as the water table was approximately 6 m below the surface. The upper boundary condition was set at “atmospheric” with surface runoff.

The Van Genuchten parameters (Θ_r_, Θ_s_, α, n, K_s_, l) were first estimated by supplying the Rosetta database internally available in HYDRUS 1D with texture and soil density data from cylinder samples in H2 (Table 5). Subsequently, a manual sensitivity test suggested that only n and K_s_ were the decisive factors for variation in water content. Thus, these parameters were refined for both soil layers by an inverse solution using daily water content data from two capacitance sensors (Sentek Drill&Drop, Sentek Sensor Technologies (2020)) installed in H2, which is adjacent to H3 and H4 and has a similar soil type. The data considered for the calibration was from 10 cm depth for the first Sentek instrument, from 50/70/100/120/150 cm depth for the second one, and a time horizon from 1^st^ of January to 14^th^ of April 2019 (104 days). Thus, winter and spring months were covered, when soil cover, plant growth, plant transpiration, and plant water uptake were negligible. Differences in water uptake among crops and root effects were not considered.

**Table 3:**
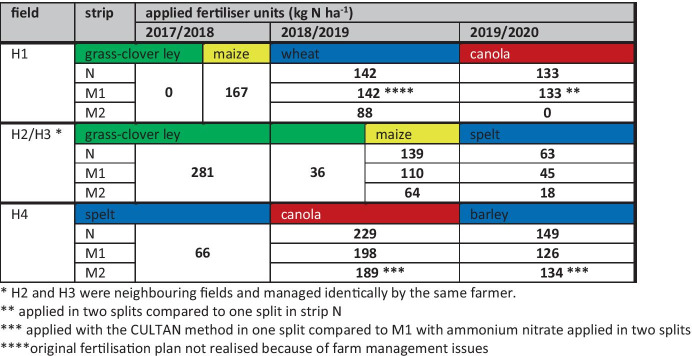
Fertilisation on the experimental fields H1, H2/3, and H4. From 2019 onwards, normal fertilisation (N) and mitigation measures (M1 and M2) were implemented on separate strips. Where organic fertiliser was used, total N rather than available N was taken into account

**Table 4:** Overview of the specifications of the monitoring techniques used in this study

	**SIA**	**Nmin soil coring**	**Suction Cups**
Description	**flux** of leached N	Nmin **content** in the soil	**concentration** of leached N
Unit	kg N ha^−1^ period^−1^	kg N ha^−1^	mg N L^−1^
Temporal resolution	Yearly	2 × /year	Monthly
Temporal specification	Time-integrated	Snapshot	Time-averaged
Comments	-	Autumn value can be interpreted as leaching potential	Conversion to [kg N ha^−1^] with water flux model

### Statistical analysis

All data management and processing were done in R Studio (version 1.3.1056). Linear regression was calculated for data comparison and statistics. The significance level α was generally set at 0.05. The coefficient of determination (R^2^) and the 95% confidence interval for the slope (m) and the intercept on the y-axis (q) were calculated. Where the data set allowed it, the standard error of the mean was computed.

For the SIA data, an analysis of variance was calculated with the *stats* package considering the field, the year, and the interaction between them. For the comparison of SIA leaching fluxes between fields, the TukeyHSD post hoc test was used for the evaluation of pair means. Generally, the logarithm of the SIA flux measurement was taken to fulfil the underlying assumptions of homoscedasticity and normality of residuals.

The statistical index “root mean square error” (RMSE) was used to assess the goodness of fit of the water flux model (Doltra & Muñoz, 2010; Willmott, 1982). This index shows the average difference between modelled (M_i_) and observed values (O_i_) among n pairs. It is computed as follows:

$$\mathbf{R}\mathbf{M}\mathbf{S}\mathbf{E}=\sqrt{\frac{{\sum }_{\mathbf{i}}^{\mathbf{n}}{({\mathbf{O}}_{\mathbf{i}}-{\mathbf{M}}_{\mathbf{i}})}^{2}}{\mathbf{n}}}$$

## Results

### SIA measurements

**Fig. 2:**
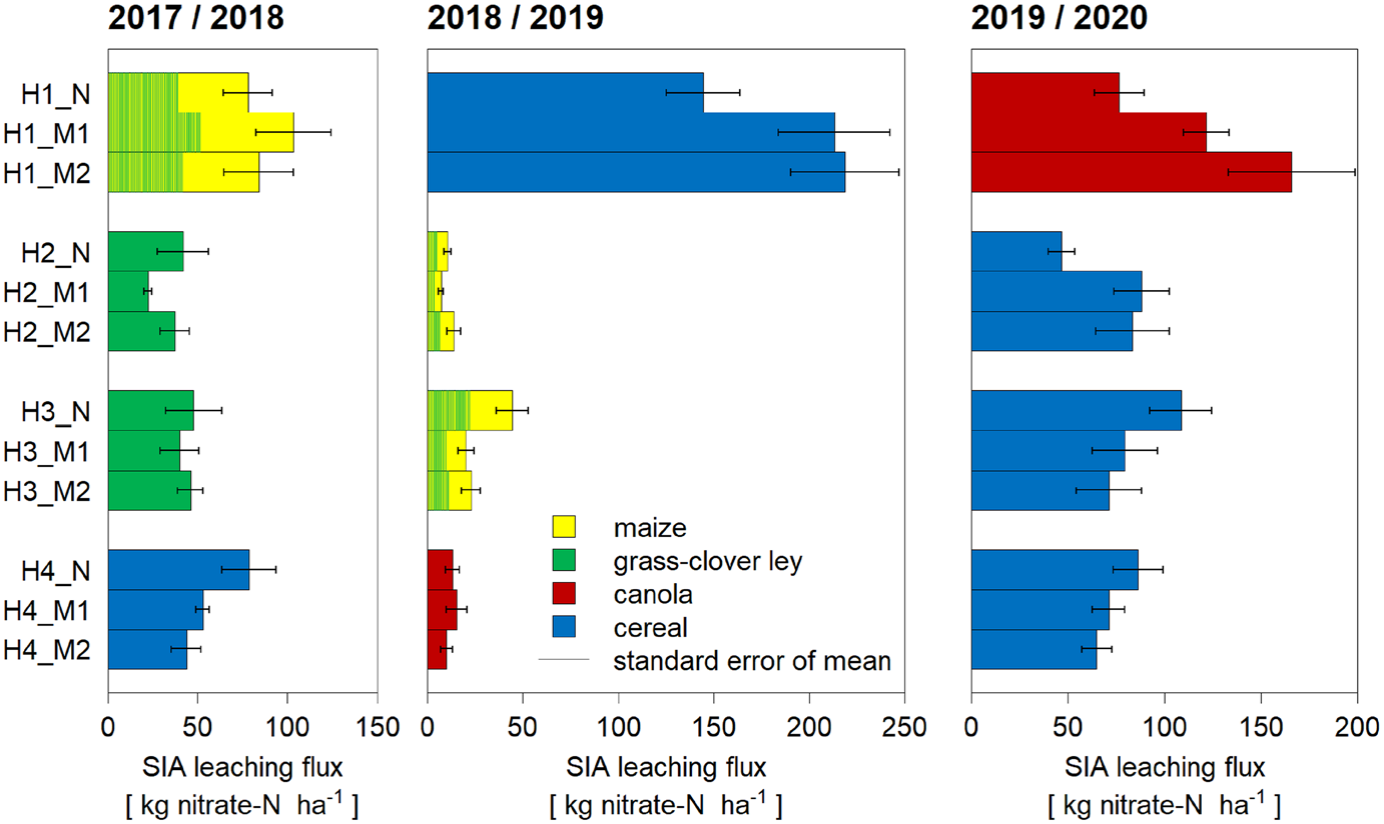
Nitrate leaching fluxes for the SIA method for each strip and per crop for the periods 2017/2018, 2018/2019 and 2019/2020. Error bars indicate standard error of the mean. The colour of the column illustrates the main crop during a given period, with the transition from grass-clover ley to maize shown by a mixed pattern with both colours. Mitigation strategies were implemented only from April 2019 onwards (Table 3)

The field H1 showed significantly higher SIA leaching fluxes than the other three fields (Fig. 2). Generally, the statistical analysis showed that the specific year, field and the interaction of these two variables significantly affected SIA fluxes. The highest annual fluxes were observed under cereal crops (44–219 kg N ha^−1^), while fluxes under the other crops varied between fields and years. For example, nitrate leaching under canola ranged from 77 to 166 kg N ha^−1^ in H1 in the third year of the study, while it was only about 15 kg N ha^−1^ in H4 in the second year.

The minimum and the maximum leaching flux both occurred in the measurement period 2018/2019, with 7 and 219 kg NO_3_^−^-N ha^−1^ in H2_M1 and H1_M2, respectively. The two neighbouring fields H2 and H3 showed similar leaching fluxes under grass-clover ley (2017/18) and subsequently under maize (2018/19). In the following year under cereal, the leaching in H2_N was much smaller than in H3_N (47 versus 109 kg NO_3_^−^-N ha^−1^) and smaller than the observed values in strips M1 and M2 with reduced fertiliser application.

Also, in other cases, the applied mitigation strategies did not show the targeted reduction in NO_3_^−^ leaching. For example, though no fertiliser was applied to H1_M2 in 2019/2020 to canola (Table 3), leaching in this strip was higher than in the N part with the farmer’s usual fertilisation (166 versus 77 kg NO_3_^−^-N ha^−1^). However, this pattern had already been visible the year before, when the strips had been fertilised equally.

### Nmin measurements

**Fig. 3:**
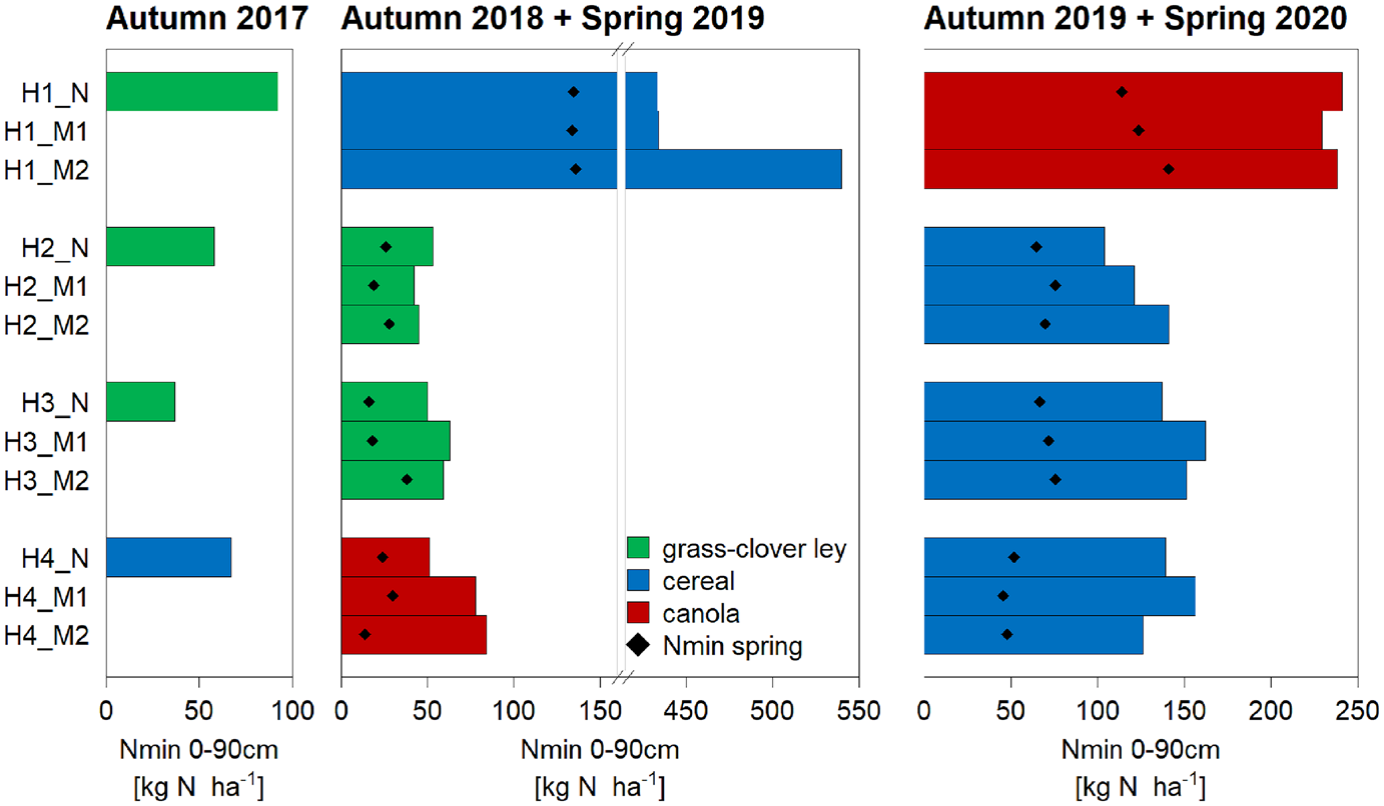
Nmin content of the soil layer 0–90 cm per strip and sampling campaign. Autumn values are displayed with bars, while diamonds indicate the corresponding Nmin value in the following spring. The actual crop at sampling time is shown in colour

As expected, Nmin values in a given strip were consistently higher in October than in the following February, thus after the winter leaching period (Fig. 3). Except for H1, autumn and spring values were lower in 2018/2019 than in 2019/2020, with the mean Nmin level (without H1) in spring 2019 being about 24 kg N ha^−1^, and in spring 2020 around 64 kg N ha^−1^. The difference between autumn and spring (∆Nmin) was larger in 2019/2020 than in 2018/2019.

As for the SIA, field H1 had generally higher values than the other three fields. For example, the spring Nmin content in H1 (130 kg N ha^−1^) was much higher than in the other fields. This value was similar in both years, even though autumn levels were much higher in 2018. Indeed, the overall maximum total Nmin value of 540 kg N ha^−1^ was found in H1_M2 in autumn 2018.

The NO_3_^−^ mitigation strategies in strips M1 and M2, applied during the cropping season 2019 in all fields, were not reflected in the Nmin values of autumn 2019.

Autumn Nmin values were predominantly in the form of nitrate, with a mean share of ammonium of 4.6% of total Nmin. Due to these low values, ammonium is not displayed and discussed separately.

### Water flux model fit

**Fig. 4:**
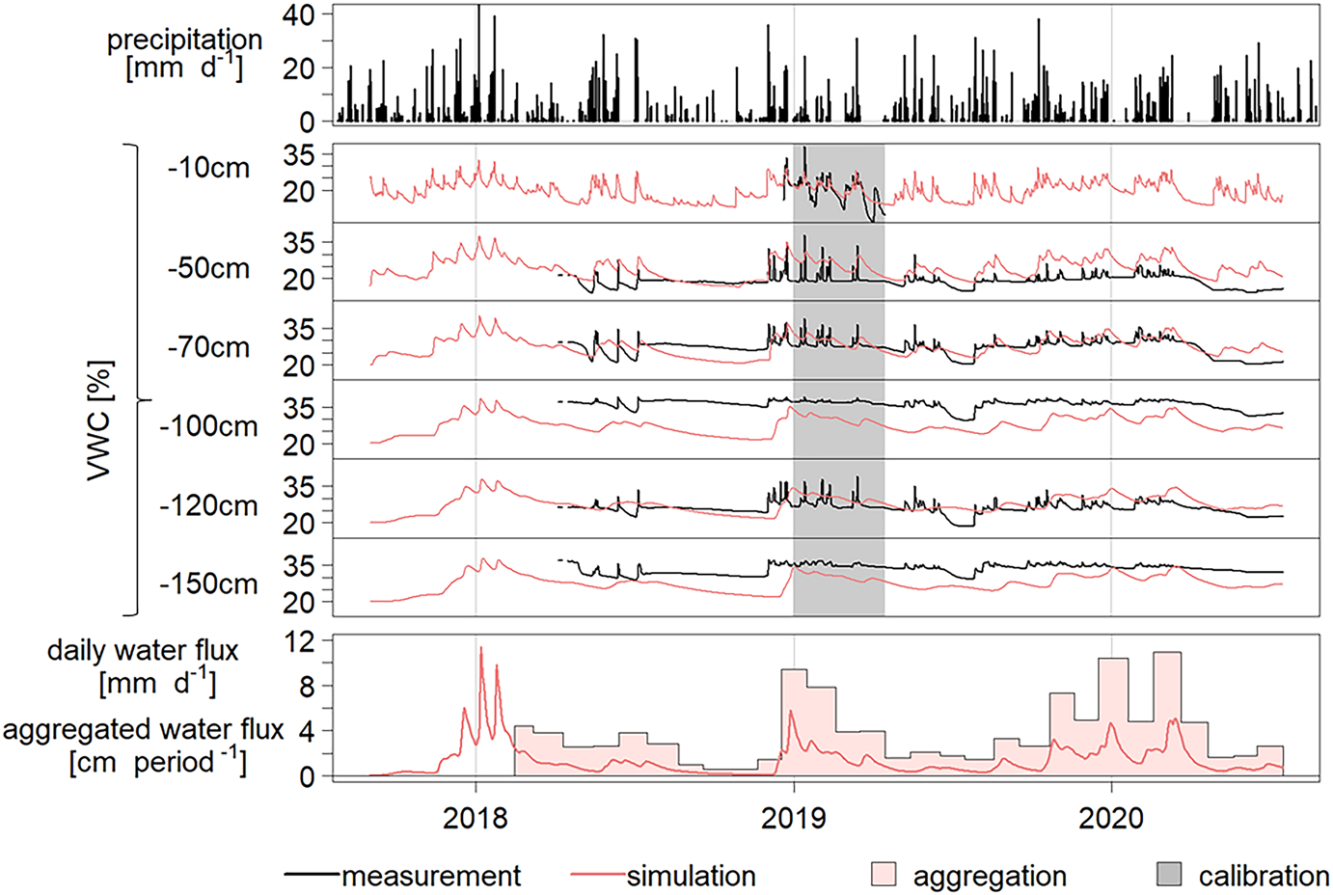
Daily precipitation values (MeteoSuisse), and measured (Sentek) and simulated (HYDRUS 1D) volumetric water contents in six depths from September 2017 to June 2020. The bottom figure shows the simulated water flux as daily values and in the aggregated form per suction cup period

By calibrating the previously estimated Van Genuchten parameters α and K_s_, a clear difference between the upper plough layer and the rest of the profile became visible, with the top layer showing a higher K_s_ and higher α (Table 5). The simulated water content (WC) visually followed the measured WC pattern in all six depths (Fig. 4). The Root Mean Square Error (RMSE) was 6.7%, describing the average deviation from measured values, and considering all six depth levels and data from 01.01.2018 to 16.07.2020. Water infiltration was seen after precipitation events and especially during the winter months (Fig. 4, Table 6).

### Suction cups measurements

**Fig. 5:**
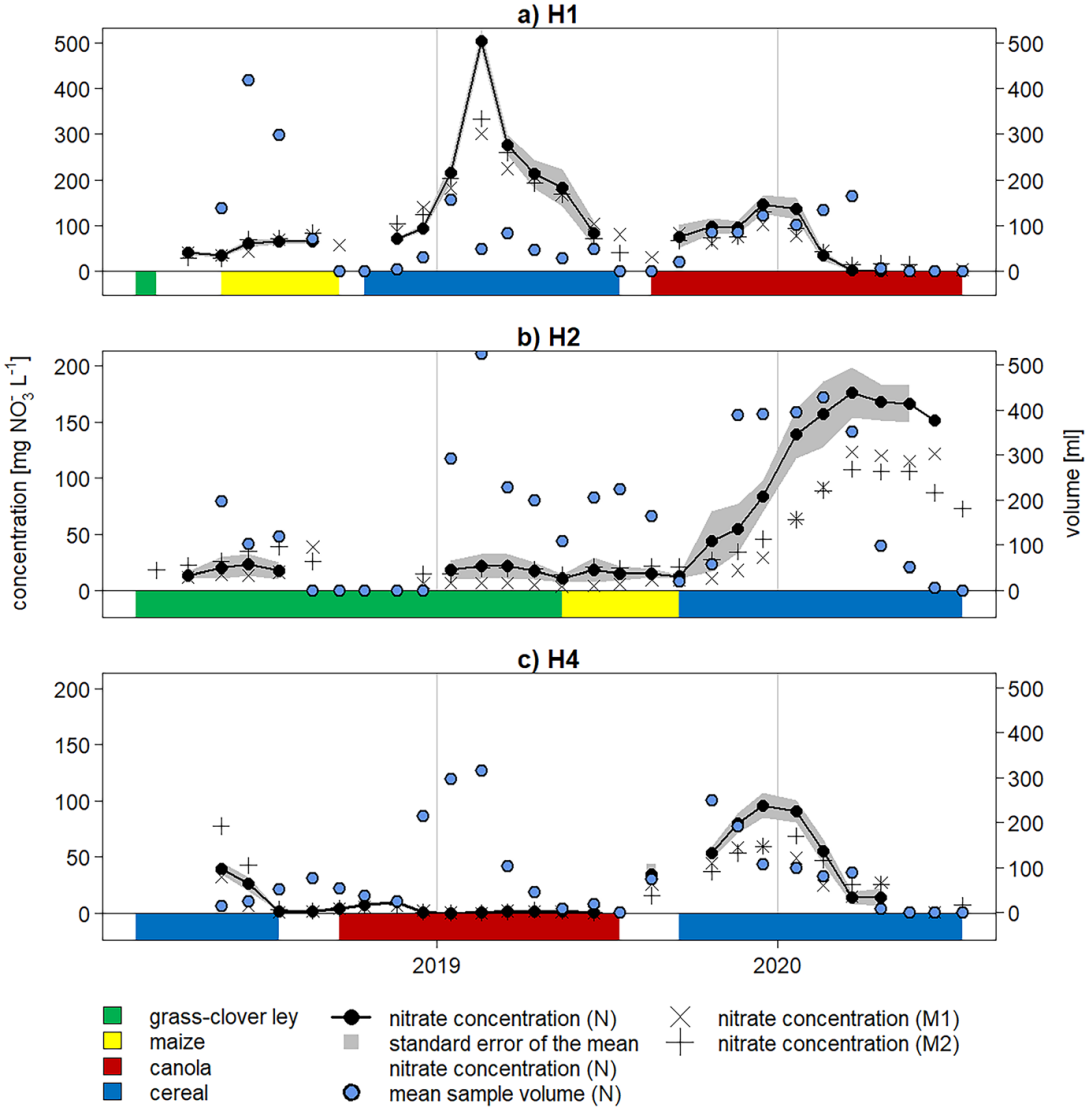
Nitrate concentrations and volume of water extracted from suction cups for the neutral strips (N) of fields H1, H2 and H4. The standard error of the mean is given as grey background. Note the different scale of the first y-axis for H1

The extracted water volume per sampling campaign and the NO_3_^−^ concentrations showed large variability with time and field (Fig. 5). For all fields, there were dry periods when no samples were extracted, mostly in summer.

The ammonium concentration was below the detection limit in most cases, except for a few cases when ammonium levels rose to 0.5 mg L^−1^. Due to these overall low values, we neglected N coming from ammonium for the SC data and present only nitrate concentrations.

Nitrate peaks typically showed a build-up and decline over several months. The largest peak in NO_3_^−^ concentration was seen in H1_N in winter 2019 after the maize harvest, reaching almost 500 mg NO_3_^−^ L^−1^. In the other two strips (M1 and M2), this peak reached about 300 mg NO_3_^−^ L^1^ each. Simultaneously, NO_3_^−^ concentrations in the other two fields remained stable at a relatively low level. In H4_N, the concentration even dropped to values close to zero, while the mean extracted water volume was rising.

Additional concentration peaks were visible in winter 2019/2020 in all fields, with 200 mg NO_3_^−^ L^−1^ in H2_N and around 150 mg NO_3_^−^ L^−1^ in H1_N and H4_N. Thus, a similar concentration pattern with high NO_3_^−^ concentrations after maize harvest was observed in H2_N and H1_N, which had an identical crop rotation shifted by one year. After the canola harvest (H4_N), the increase in concentration was somewhat smaller.

**Fig. 6:**
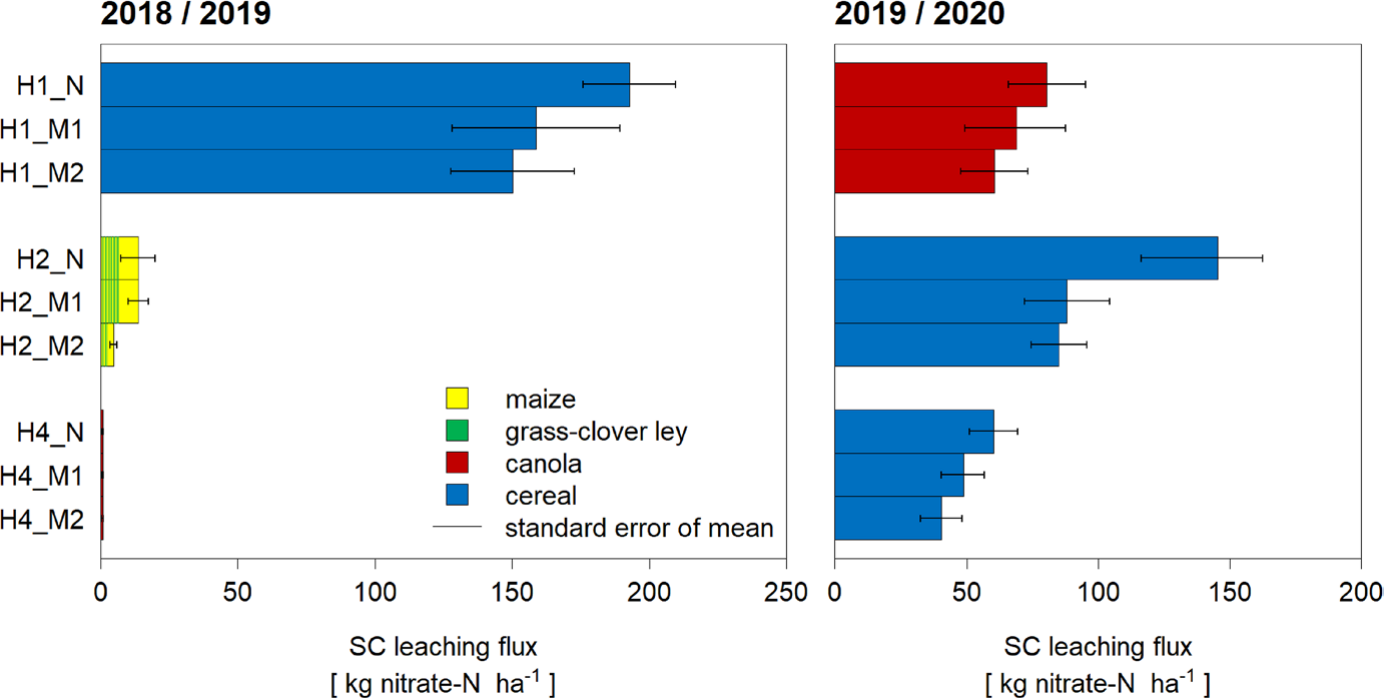
Nitrate leaching as measured by suction cups and converted into kg N ha^−1^ per period and strip using simulated water leaching fluxes. The standard error was derived from SC concentrations. As SCs were installed only in spring 2018, it was not possible to calculate a SC leaching for the entire period 2017/2018

These concentration patterns were reflected in the calculated leaching fluxes, i.e., multiplying the SC concentrations with the simulated water fluxes (Fig. 6). Aggregated to an entire cropping period, the build-up under cereal in H1_N transforms into a leaching loss of 193 kg NO_3_^−^-N ha^−1^, whereas in H2 and especially H4, the losses were much lower. In 2019/2020, the losses were more balanced among fields. Looking at the fields under cereals, the losses were smaller in H2 and H4 than in the previous year in H1. In all fields, the strip with the highest fertilisation (N) consistently showed the highest nitrate loss (Fig. 6).

Additionally, the flow-weighted nitrate concentrations for the periods 2018/2019 and 2019/2020 were calculated (Supplementary Information).

## Discussion

**Fig. 7:**
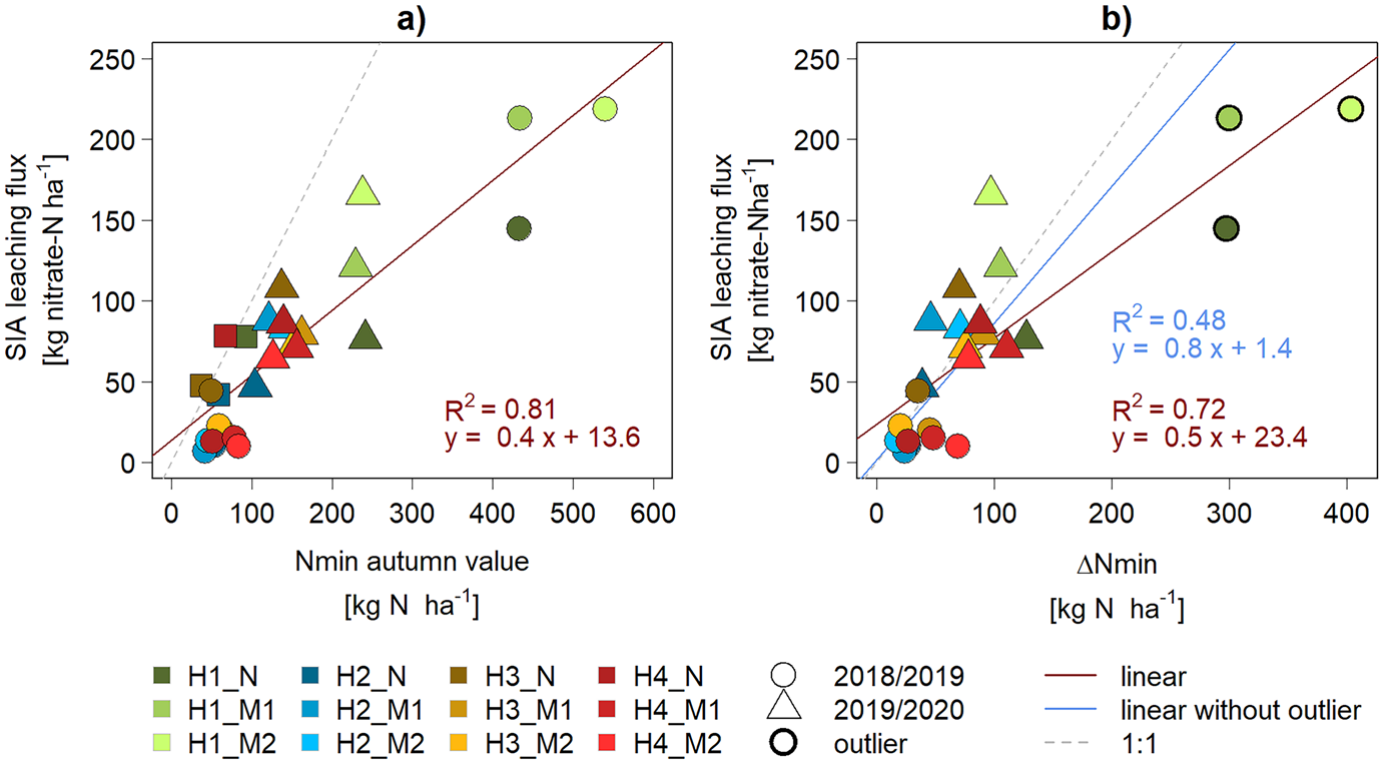
Comparison between the Nmin and SIA datasets. ∆Nmin refers to the difference between spring and autumn Nmin values

**Fig. 8:**
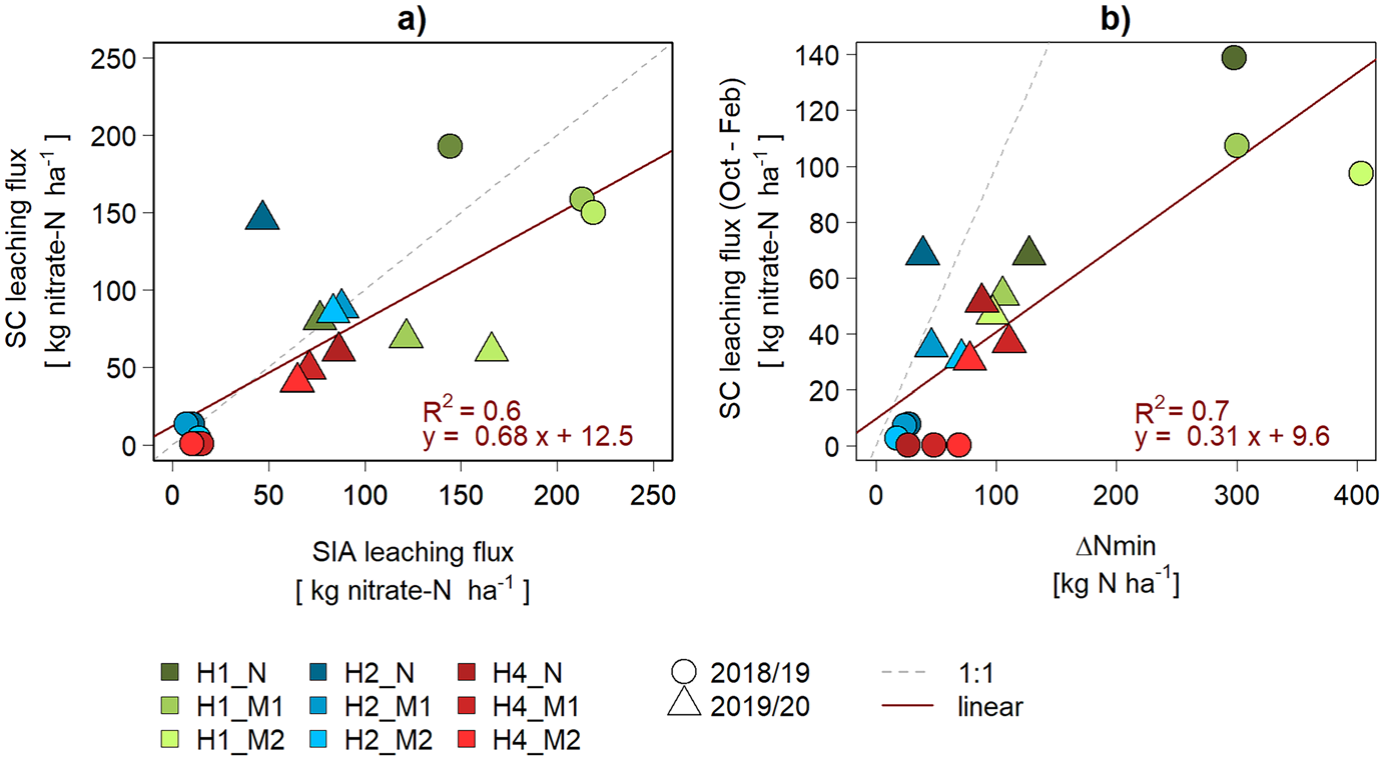
Comparison of the SC dataset with SIA and Nmin data. **a** Comparison between the directly measured SIA and the computed SC leaching fluxes. **b** Comparison of ∆Nmin and the SC leaching fluxes in the same period (October to February). Years are indicated by the shape, fields and strips by the colour of the symbol

The three in situ methods to quantify nitrate leaching in arable fields generally showed positive linear correlations with each other, while absolute values differed (Fig. 7, Fig. 8). In the following sections, we first discuss the mechanistic understanding that can be derived from comparing the temporal (**“Nitrate leaching occurs mainly during winter months”**) and spatial (**“Preferential flow is an important leaching factor”**) variation of the different approaches before concluding with an overall assessment on practical aspects (**“The choice of methods depends mainly on project goals”**) and data quality (**“Long-term datasets are essential”**).

### Nitrate leaching occurs mainly during winter months

The monthly SC nitrate concentrations showed a seasonal pattern (Fig. 5). The build-up during autumn and a peak in winter suggest a main nitrate loss between October and February. Initially, we approximated the extent of this winter leaching by ∆Nmin, i.e., the difference between Nmin values in autumn and in the following spring. However, this procedure assumes no significant N loss other than leaching during the winter months. The approximation indicated that 70% of the autumn content above a specific amount of 19.4 kg N ha^−1^ was lost (m = [0.65, 0.79], q = [− 33.9, − 5.00]). These values are similar to a study on farmers’ fields in the Czech Republic, where a winter loss of 74% of autumn Nmin measured in 0–60 cm was observed above 25 kg N ha^−1^ (Haberle et al., 2009).

When plotting the Nmin autumn value against the leaching measured with SIA devices, the percentage of autumn Nmin lost by leaching in the following season was 40% (m = [0.32, 0.48], R^2^ = 0.81 in Fig. 7a). As the ∆Nmin period only includes 4r months (mid-October to mid-February) compared to a full agricultural season in the SIA method, it was expected that ∆Nmin values would be smaller than the SIA leaching fluxes. Surprisingly, this comparison (Fig. 7b) showed the opposite trend, especially for three outliers in 2018/2019 (H1). The monthly SC data allowed us to approximate losses between autumn and spring Nmin by aggregating SC fluxes between October and February, even though SC and Nmin sampling dates did not coincide completely but were generally a few days apart. The comparison between the aggregated SC flux and ∆Nmin indicates that 30% of ∆Nmin was detected as SC leaching (Fig. 8b).

Together, these observations indicate additional N sinks within the measurement period of ∆Nmin, e.g., incorporation of Nmin into the plant and/or microbial biomass in late autumn or gaseous loss (N_2_O and N_2_) due to denitrification (Ramos & Kucke, 2001). The dry meteorological conditions would not facilitate major denitrification processes. The high autumn temperatures (mean values of above 10 °C in October 2017–2019) support the hypothesis of late N incorporation, as microbial N cycling and plant growth continue after the autumn sampling. In H1, high Corg values could have further promoted late N assimilation (Table 2). Thus, the required conditions for a proper Nmin autumn campaign, namely low temperatures while winter precipitation has not yet started, were only partially met due to exceptionally high temperatures during the study time (Osterburg et al., 2007; Niedersächsischer Landesbetrieb für Wasserwirtschaft, 2012).

### Preferential flow is an important leaching factor

The comparison of the directly measured SIA leaching fluxes (Fig. 2) with the ones derived from SC concentrations and simulated water leaching fluxes (Fig. 6) suggests a linear relationship (Fig. 8a), with a tendency for higher SIA compared to SC leaching fluxes (m = [0.39, 0.98]). The SIA devices probably partially measured preferential flow, which also explains the large standard error of the means (Fig. 2). Preferential flow is represented in SC concentrations only to a limited extent, as they mainly sample from the soil matrix, which leads to selective sampling (Barbee & Brown, 1986; Grossmann & Udluft, 1991; Webster et al., 1993; Fares et al., 2009; Wang et al., 2012, Singh et al., 2017). Specifically, the ceramic cup material is of a very specified pore size, which determines the suction limit. Consequently, only a small range of pores can be sampled effectively, i.e. those close to the same suction as the cup material. In fast flows, the hydraulic conductivity in the SCs is too low. Also, the connectivity to the surrounding soil medium is usually not complete. Preferential flow in soil cracks, earthworm and root channels (macropores) is therefore not represented in the SC samples, as during percolation events, the cup does not take up the fast-moving water. On the other hand, the suction of the SCs is too low to capture slow flow, e.g. in inter-clay pores. In brief, fast and slow flows are underestimated with the SC method.

Preferential flow might be responsible for 1/3 of the total leaching flux, visible in the regression slope in Fig. 8a. In other words, leaching increases by 50% when preferential flow is considered in addition to matrix flow, but with large differences among fields. In simulations of Larsson and Jarvis (1999), N leaching in clay soil in southwest Sweden was 34% higher with macropore transport. However, the authors emphasise that this observation is only valid for short-term data. For longer periods, macropore flow even reduced leaching, as, during winter, the NO_3_^−^ concentration in the bypassing water is lower than in the soil water.

### The choice of methods depends mainly on project goals

Project goals should inform the choice of the monitoring methods. Factors such as the prevalent soil type, the extent of the catchment, previous knowledge about regional hydrological processes and agricultural management, as well as financial and temporal constraints of the research project must be taken into account (Table 7).

**Table 5:** Soil hydrological parameters of the HYDRUS 1D model using the Rosetta database and a calibration

	Field parameters				Averaged Van Genuchten parameters according to Rosetta database					Matrix parameters after calibration	
Depth [cm]	Clay [%]	Silt [%]	Sand [%]	Density [g cm^−1^]	Θr [-]	Θs [-]	α [cm^−1^]	n [-]	K_s_ [cm d^−1^]	α [cm^−1^]	K_s_ [cm d^−1^]
0–30	11	54	36	1.68	0.0576	0.4701	0.0039	1.7405	177.8	0.041	200.0
30–60	10	53	37	1.76						0.024	12.7
60–150	13	61	26	1.78

**Table 6:** Monthly and yearly measured precipitation data (MeteoSuisse) and percolation values simulated with HYDRUS 1D for the years 2018, 2019 and 2020. For comparison, the precipitation norm data for the Wynau station for 1961–1980 is given (MeteoSuisse), as well as the estimated direct groundwater recharge in the Gäu based on tracer experiments (Gerber et al., 2018)

	For comparison		2018		2019		2020	
	Precipitation [mm] *	Percolation [mm] **	Precipitation [mm]	Percolation [mm]	Precipitation [mm]	Percolation [mm]	Precipitation [mm]	Percolation [mm]
January	76	-	199	185	73	89	41	71
February	72	-	52	81	39	56	135	59
March	70	-	75	41	90	41	77	110
April	69	-	16	29	22	28	32	27
May	95	-	177	17	133	15	86	19
June	108	-	70	38	58	22	138	21
July	94	-	107	33	103	16	36	23
August	104	-	48	17	132	22	128	13
September	79	-	34	9	54	28	60	15
October	76	-	31	6	145	44	123	33
November	84	-	20	5	77	67	32	67
December	87	-	190	58	115	77	89	45
Annual sum	1013	380—460	1017	519	1040	504	975	503

* Precipitation norm data from Wynau station for 1961–1980 (MeteoSuisse)

** Estimated direct groundwater recharge in the Gäu valley based on tracer experiments (Gerber et al., 2018)

**Table 7:** Advantages, difficulties and limitations of the three monitoring methods

	**Self-Integrating Accumulators (SIA)**	**Nmin soil coring (Nmin)**	**Suction Cups (SCs)**
Possible scientific goal	- Comparison of several fields by crop or year - Comparison of strips with leaching mitigation strategies - Identification of the fertiliser fraction that is lost	- Identification of residual N in autumn as indicator of loss potential, e.g. with mitigation strategies - Estimation of winter loss - Spring Nmin value for adjustment of fertilisation	- Comparison with the legal nitrate concentration target in groundwater - Identification of hot moments of leaching during the year - Combination with water leaching models to identify N loss flux
Advantages	- Result is area-related - Upscaling to the field and comparison with N input is feasible - Preferential flow is taken into account	- Result is area-related - Upscaling to the field and comparison with N input is feasible	- Preparation of the samples only includes filtration - Nitrate concentration is comparable to legal groundwater values - Using Ion Chromatography, information on all anions and cations become available
Difficulties	-	- Ideal sampling date in autumn is delicate as it depends on temperature and rainfall	- Careful installation needed to ensure direct soil contact of the cups - Unstable vacuum may occur because of a leaking tubing system - Limited information on water and N fluxes: A soil model is needed - Preferential flow is only partially captured, as the cups take the water from the soil matrix
Problematic factors for application	Upwelling soil water (stagnic soil properties)	High stone content in the soil profile	
Spatial resolution	Middle – high (versatile)	High (versatile)	Low
Temporal resolution	Low	Low – middle	High
Initial costs and time	Low	Low	High
Returning time per strip (without transport)	12 h/field/year	12 h/field/year	30 h/field/year
Sample preparation before analysis	Homogenisation + extraction of SIA material	Sieving, homogenisation and extraction of soil samples	Filtering of liquid samples
Dismantling costs	Low	None	High

Not every method can be used on every site. For example, a high stone content can bias the results of Nmin soil coring, as field samples at these specific spots can hardly or not at all be collected (Niedersächsischer Landesbetrieb für Wasserwirtschaft, 2012). When soil water is moving upwards, e.g. with a high water table, the use of SIA devices and SCs is not recommended, as nitrate from lower soil layers may end up in the instruments. Therefore, the soil profile must be examined for reducing properties before installation. Historical drawings of river systems, official pedological maps, and farmers’ knowledge can help to find current and past water accumulation trends in the subsurface. Additionally, stagnant water induces soil denitrification, and thus the analysis of NO_3_^−^ as the only target component would generally not adequately picture the N loss processes.

Temporal dynamics can only be identified with SCs, thanks to the method’s high temporal resolution (Table 7). Knowing the seasonal timing of leaching events is helpful when process understanding is needed, e.g., for identifying mitigation strategies of maximum efficacy. Also, SC concentrations from below the root zone can directly be compared to groundwater legislation specifying a nitrate concentration limit. A soil model calibrated with WC measurements can complement the observations for simulation of water percolation. On the other hand, the SC system has the drawback of high initial labour and material costs (Table 7). Additionally, the spatial coverage is low, as instruments have to be installed close to the field border due to physical constraints in vacuum transport.

For the monitoring and comparison of mitigation strategies, the SIA method seems to be the preferred approach, despite its lower temporal resolution. The method convinces through high and versatile spatial coverage and low material costs. On the other hand, expertise for correct material preparation and installation is required. A long-term regional dataset allows identifying the main influencing leaching factors like soil type, annual meteorology, crop, and fertilisation. The SIA leaching flux can be compared directly with fertiliser application rates, as results are area-related and can thus be spatially upscaled.

The Nmin approach is similar to the SIA method in its advantages of being low-cost and versatile regarding temporal and spatial resolution. However, the autumn method does not measure leaching but rather indicates a loss potential. In this study, autumn Nmin and actual leaching showed a satisfying relationship (Fig. 7b). However, this statistical regression has to be confirmed by future data.

A combination of methodological approaches is preferred or even required to crosscheck results, detect outliers, decrease the uncertainty, and increase the general understanding of N cycling. However, parallel monitoring might exceed financial budgets in the long term. Therefore, we suggest using a simple method, like the Nmin autumn content, as an indicator after establishing a relationship with a more sophisticated approach, e.g. with SCs or SIA devices.

### Long-term datasets are essential

Multiannual data are required for assessing nitrate leaching under agricultural fields due to meteorological irregularities, crop rotations of several years’ extent, and a time lag between activity on the surface (e.g. the N fertilisation) and a visible leaching effect. This study illustrated this necessity by the nitrate mitigation strategies implemented in the seasons 2019/2020 (Table 3). The reduced fertiliser quantities had limited influence on the measured leaching, e.g., in the Nmin autumn values of 2019, changes in SC concentrations or SIA leaching fluxes. In these cases, we assume that the stock of soil organic N was large enough to ensure continuous mineralisation and, consequently, high soil nitrate levels. For example, we observed high leaching concentrations in the winters following a ley termination, which probably increased the turnover of soil organic matter, explaining the strong accumulation of nitrate in the root zone.

A long-term tracer study with isotopically labelled N fertiliser in lysimeters showed that, after three decades, 12–15% of applied N was still incorporated in soil organic matter (Sebilo et al., 2013). Thus, the overall time lag for the N transfer from the soil surface to a drinking water well does not only consist of the delay regarding transport in the vadose zone and the aquifer. This N must first be transformed into nitrate, mainly produced from soil organic matter through mineralisation processes (Kendall & McDonnell, 1998). The NO_3_^−^ release rate is difficult to estimate because of the wide variety of fertiliser compositions and dependencies on environmental factors like temperature and soil humidity (Di & Cameron, 2002). Nitrate is only leached when its accumulation in the soil coincides or is followed by precipitation values large enough to cause water percolation (Di & Cameron, 2002). Our SC data and the water flux simulation show that this happens during wintertime, which is several months after implementing mitigation strategies.

With the present dataset, we cannot yet fully assess the efficacy of the nitrate leaching mitigation strategies, but we have shown that mitigation strategies can be evaluated with any of the tested methods. The agronomic recommendations of our data will be discussed in a consecutive paper with a longer time series.

## Conclusions

This study has shown that not all leaching processes are equally represented by the monitoring methods used here (Suction Cups, Nmin soil sampling and SIA devices). Based on the findings, no unique single method can be used as a benchmark. A combination of methodological approaches is thus preferred to picture the influence of winter precipitation events, preferential flow mechanisms, and a continuous N cycling with elevated temperatures in late autumn on nitrate leaching. In general, the combination of methods generates additional knowledge beyond the methodological comparison.

Additionally, it is essential to get a multiannual dataset. Data from a single season is not sufficient due to meteorological irregularities, crop rotations of several years’ extent and temporal delays regarding N application, N cycling and nitrate leaching. However, parallel monitoring might exceed financial budgets in the long term. Therefore, we suggest using a simple method, like the Nmin autumn content, as an indicator, after having established a good relationship with a more sophisticated approach like SC or SIA.

This study identified high financial and labour costs for all monitoring techniques of nitrate leaching in the soil. It would be a significant step forward to be able to track nitrate with an in-situ sensor in the field soils, including real-time data transfer.

## Supplementary Information

Below is the link to the electronic supplementary material.

Supplementary file1 (DOCX 144 KB)

## Data Availability

All data generated or analysed during this study are included in this published article and its supplementary information files. In case more data is needed for specific purposes, it is available from the corresponding author on request.

## References

[CR1] Abdou HM, Flury M (2004). Simulation of water flow and solute transport in free-drainage lysimeters and field soils with heterogeneous structures. European Journal of Soil Science.

[CR2] Adesemoye AO, Torbert HA, Kloepper JW (2008). Enhanced plant nutrient use efficiency with PGPR and AMF in an integrated nutrient management system. Canadian Journal of Microbiology.

[CR3] Agroscope. (1996). Méthodes de référence des stations fédérales de recherches agronomiques. Analyse de terre pour conseil de fumure. Extraction du NO3-N et de l'NH4-N par le chlorure de calcium 0.01M (1:4) pour déterminder la teneur en Nmin.

[CR4] Anger M (2002). Nitrat-Austräge auf intensiv und extensiv beweidetem Grünland, erfasst mittels Saugkerzen- und Nmin-Beprobung - Variabilität der NO3- und NH4-Werte und Aussagegenauigkeit der Messmethoden. Journal of Plant Nutrition and Soil Science.

[CR5] Barbee GC, Brown KW (1986). Comparison between suction and free-drainage soil solution samplers. Soil Science.

[CR6] Bischoff W-A (2007). Development and applications of the self-integrating accumulators: A method to quantify the leaching losses of environmentally relevant substances.

[CR7] Böhlke J-K (2002). Groundwater recharge and agricultural contamination. Hydrogeology Journal.

[CR8] Cameron KC, Di HJ, Moir JL (2013). Nitrogen losses from the soil/plant system: A review. Annals of Applied Biology.

[CR9] Di HJ, Cameron KC (2002). Nitrate leaching in temperate agroecosystems: Sources, factors and mitigating strategies. Nutrient Cycling in Agroecosystems.

[CR10] Doltra J, Muñoz P (2010). Simulation of nitrogen leaching from a fertigated crop rotation in a Mediterranean climate using the EU-Rotate_N and Hydrus-2D models. Agricultural Water Management.

[CR11] EU Commission. (1991). Directive 91/676/EEC. Council Directive of 12 December 1991 concerning the protection of waters against pollution caused by nitrates from agricultural sources. L375. *Official Journal of European Community,* 1–8.

[CR12] European Environment Agency. (2018). European waters — Assessment of status and pressures 2018. EEA Report Luxembourg.

[CR13] Fares A, Deb SK, Fares S (2009). Review of vadose zone soil solution sampling techniques. Environmental Reviews.

[CR14] Gabriel, O., Brielmann, H., Humer, F., & Grath, J. (2016). Drainagemonitoring - eine ergänzende Methode zur Evaluierung von Maßnahmenwirksamkeiten zur Reduktion von Nitratemissionen. 5. Umweltökologisches Symposium 2016, Höhere Bundeslehr- und Forschungsanstalt Raumberg-Gumpenstein, 57–64.

[CR15] Gerber C, Purtschert R, Hunkeler D, Hug R, Sültenfuss J (2018). Using environmental tracers to determine the relative importance of travel times in the unsaturated and saturated zones for the delay of nitrate reduction measures. Journal of Hydrology.

[CR16] Grossmann J, Udluft P (1991). The extraction of soil water by the suction-cup method: A review. Journal of Soil Science.

[CR17] Haberle J, Kusá H, Svoboda P, Klir J (2009). The changes of soil mineral nitrogen observed on farms between autumn and spring and modelled with a simple leaching equation. Soil and Water Resources.

[CR18] Hendrickx, J. M. H., Corwin D. L., & KachanoskiI, R. G. (2002). Chapter 6: Miscible solute transport. *Methods of Soil Analysis, Part 4.*

[CR19] Hunkeler, D., Sonney, R., Paratte, D., Tallon, L., & Gerber, C. (2015). Nitratprojekt Gäu-Olten: Hydrochemische Erkundung des Grundwasserleiters und Bestimmung der Altersstruktur.

[CR20] IUSS Working Group WRB. (2015). World reference base for soil resources 2014 — International soil classification system for naming soils and creating legends for soil maps. *World Soil Resources Report.* Rome, FAO.

[CR21] Jabloun M, Schelde K, Tao F, Olesen JE (2015). Effect of temperature and precipitation on nitrate leaching from organic cereal cropping systems in Denmark. European Journal of Agronomy.

[CR22] Kantonales Amt für Umwelt (AfU) Solothurn. (2020). Nitratkonzentration im Pumpwerk Neufeld.

[CR23] Kendall C, McDonnell JJ (1998). Isotope tracers in catchment hydrology.

[CR24] Klages, S., Surdyk, N., Christophoridis, C., Hansen, B., Heidecke, C., Henriot, A., Kim, H., & Schimmelpfennig, S. (2018). Review report of AgriDrinking water quality indicators and IT/sensor techniques, on farm level, study site and drinking water source, FAIRWAY.

[CR25] Knittel, H., Albert, E., & Ebertseder, T. (2012). Praxishandbuch Dünger und Düngung, Agrimedia.

[CR26] Larsson MH, Jarvis NJ (1999). A dual-porosity model to quantify macropore flow effects on nitrate leaching. Journal of Environmental Quality.

[CR27] Li ZM, Skogley EO, Ferguson AH (1993). Resin adsorption for describing bromide transport in soil under continuous or intermittent unsaturated water flow. Journal of Environmental Quality.

[CR28] McClain ME, Boyer EW, Dent CL, Gergel SE, Grimm NB, Groffman PM, Hart SC, Harvey JW, Johnston CA, Mayorga E, McDowell WH, Pinay G (2003). Biogeochemical hot spots and hot moments at the interface of terrestrial and aquatic ecosystems. Ecosystems.

[CR29] Niedersächsischer Landesbetrieb für Wasserwirtschaft. (2012). Grundwasser: Untersuchung des mineralischen Stickstoffs im Boden - Empfehlungen zur Nutzung der Herbst-Nmin-Methode für die Erfolgskontrolle und zur Prognose der Sickerwassergüte. Niedersachsen.

[CR30] Osterburg, B., Rühling, I., Runge, T., Schmidt, T. G., Seidel, K., Antony, F., Gödecke, B., & Witt-Altfelder, P. (2007). Kosteneffiziente Massnahmenkombinationen nach Wasserrahmenrichtlinie zur Nitratreduktion in der Landwirtschaft. Braunschweig, Bund/Länder Arbeitsgemeinschaft Wasser.

[CR31] Pasquier F (1986). Hydrodynamique de la nappe du Gäu (cantons de Soleure et Berne).

[CR32] Ramos, C. & Kucke, M. (2001). A review of methods for nitrate leaching measurement. Proceedings of the international conference on environmental problems associated with nitrogen fertilisation of field-grown vegetable crops. C. R. Rahn, R. D. Lillywhite, S. DeNeve, M. Fink and C. Ramos.

[CR33] Richner, W., Sinaj, S., Carlen, C., Flisch, R., Gilli, C., Huguenin-Elie, O., Kuster, T., Latsch, A., Mayer, J., Neuweiler, R., & Spring, J.-L. (2017). Grundlagen für die Düngung landwirtschaftlicher Kulturen in der Schweiz (GRUD 2017). Agroscope Schweiz.

[CR34] Rockstrom J, Steffen W, Noone K, Persson A, Chapin FS, Lambin EF, Lenton TM, Scheffer M, Folke C, Schellnhuber HJ, Nykvist B, de Wit CA, Hughes T, van der Leeuw S, Rodhe H, Sorlin S, Snyder PK, Costanza R, Svedin U, Falkenmark M, Karlberg L, Corell RW, Fabry VJ, Hansen J, Walker B, Liverman D, Richardson K, Crutzen P, Foley JA (2009). A safe operating space for humanity. Nature.

[CR35] Sebilo M, Mayer B, Nicolardot B, Pinay G, Mariotti A (2013). Long-term fate of nitrate fertilizer in agricultural soils. Proceedings of the National Academy of Sciences.

[CR36] Sentek Sensor Technologies. (2020). Sentek Drill&Drop - Probe Manual Version 1.3 for Bluetooth probes, Series II Interface probes, and Series III probes. Stepney, South Australia.

[CR37] Šimůnek, J., Šejna, M., Saito, H., Sakai, M., & van Genuchten, M. T. (2013). The HYDRUS-1D Software Package for simulating the one-dimensional movement of water, heat, and multiple solutes in variably-saturatedmedia, University of California Riverside.

[CR38] Šimůnek, J., Šejna, M., Sato, H., Sakai, M., & van Genuchten, M. T. (2008). The HYDRUS-1D software package for simulating the one-dimensional movement of water, heat, and multiple solutes in variably-saturated media, Version 4.0, Hydrus Series 3. Department of Environmental Sciences, University of California Riverside, Riverside, CA, USA.

[CR39] Singh, G., Kaur, G., Williard, K., Schoonover, J., & Kang, J. (2017). Monitoring of water and solute transport in the vadose zone: A review. *Vadose Zone Journal, 17*(1).

[CR40] Skogley EO (1992). The universal bioavailability environment/soil test unibest. Communications in Soil Science and Plant Analysis.

[CR41] Steinshamn H, Thuen E, Bleken MA, Brenøe UT, Ekerholt G, Yri C (2004). Utilization of nitrogen (N) and phosphorus (P) in an organic dairy farming system in Norway. Agriculture, Ecosystems & Environment.

[CR42] Swisstopo. (2020). Geologieportal: Letzteiszeitliches Maximum. Retrieved May 10, 2020, from https://map.geo.admin.ch/?topic=geol&lang=de&bgLayer=ch.swisstopo.pixelkarte-grau&layers=ch.swisstopo.geologie-geocover,ch.swisstopo.geologie-eiszeit-lgm-raster&layers_opacity=0.75,1&catalogNodes=1786,1802,1787,1793&E=2630200.00&N=1235600.00&zoom=3.

[CR43] Talbot W (2016). Development of a new in situ system to measure nitrate leaching losses from winter grazed fodder beet.

[CR44] UMS GmbH. (2010). Pore water sampler (suction cup) SIC20: Manual. München.

[CR45] Umweltministerium Baden-Württemberg. (2001). Verordnung des Umweltministeriums über Schutzbestimmungen und die Gewährung von Ausgleichsleistungen in Wasser- und Quellenschutzgebieten (SchALVO).

[CR46] van der Laan M, Stirzaker RJ, Annandale JG, Bristow KL, C. C. d. Preez,  (2010). Monitoring and modelling draining and resident soil water nitrate concentrations to estimate leaching losses. Agricultural Water Management.

[CR47] Vero SE, Basu NB, Van Meter K, Richards KG, Mellander PE, Healy MG, Fenton O (2018). Review: The environmental status and implications of the nitrate time lag in Europe and North America. Hydrogeology Journal.

[CR48] Wang L, Butcher AS, Stuart ME, Gooddy DC, Bloomfield JP (2013). The nitrate time bomb: A numerical way to investigate nitrate storage and lag time in the unsaturated zone. Environmental Geochemistry and Health.

[CR49] Wang Q, Cameron K, Buchan G, Zhao L, Zhang EH, Smith N, Carrick S (2012). Comparison of lysimeters and porous ceramic cups for measuring nitrate leaching in different soil types. New Zealand Journal of Agricultural Research.

[CR50] Wang YC, Ying H, Yin YL, Zheng HF, Cui ZL (2019). Estimating soil nitrate leaching of nitrogen fertilizer from global meta-analysis. Science of the Total Environment.

[CR51] Webster CP, Shepherd MA, Goulding KWT, Lord E (1993). Comparisons of methods for measuring the leaching of mineral nitrogen from arable land. Journal of Soil Science.

[CR52] Wendland, M., Brummer, S., & Haringer, G. J. (2018). Grundwasserschonende Landbewirtschaftung in den Gebieten Hohenthann, Pfeffenhausen und Rottenburg an der Laaber. Bayern, LfL Agrarökologie.

[CR53] Willmott CJ (1982). Some comments on the evaluation of model performance. Bulletin American Meteorological Society.

[CR54] Yang JE, Skogley EO (1992). Diffusion kinetics of multinutrient accumulation by mixed-bed ion-exchange resin. Soil Science Society of America Journal.

[CR55] Zhao X, Christianson LE, Harmel D, Pittelkow CM (2016). Assessment of drainage nitrogen losses on a yield-scaled basis. Field Crops Research.

