# Supplementary material for: Field-scale monitoring of nitrate leaching in agriculture: assessment of three methods
Source: Environ Monit Assess. 2021 Dec 6;194(1):4. doi: 10.1007/s10661-021-09605-x (PMC8648662; doi:10.1007/s10661-021-09605-x)
Supplement: Supplementary file 1 — Supplementary file1 (DOCX 144 KB) [file 10661_2021_9605_MOESM1_ESM.docx]

# Supplementary material

Table 8: Details and additional information for planning a nitrate monitoring campaign.

|  | **Self-Integrating Accumulators (SIA)** | **Nmin soil coring (Nmin)** | **Suction Cups (SCs)** |
| --- | --- | --- | --- |
| sampling depth  (in this project) | 1 m | 0 – 0.9 m  (0-0.3 m / 0.3-0.6 m / 0.6-0.9 m) | 1.2 m |
| spatial resolution | middle - high  - this project: three SIA pits with 4 devices each covered a third to half of the strip  - future projects: possibility to spread pits evenly along the entire strip length | high  - this project: 10 sampling points distributed along a trajectory on the entire strip | low  - installation close to the field border due to physical restrictions in vacuum transport in the piping system  - this project: 8 SCs per strip |
| temporal resolution | low  - this project: yearly exchange of SIA devices  - possibility to decrease the period | low – middle  - this project: two campaigns per year  - sampling frequency is adaptable | high  monthly sampling due to  - volume capacity of the bottles  - limited lifetime of the two 12V-batteries for the vacuum pump |
| special field material used | Material per campaign:  - excavator  - prepared SIA devices (sand + adsorber)  - GPS instrument or similar device to document location of pits | Material per campaign:  - automated auger with GPS  - cooling boxes with plastic bags and impermeable sample labels | Initial material:  - suction cups with tubes  - concrete shaft or panel  - protection pipes  - 12V batteries  - vacuum pump  - glass bottles including water stopper  - GPS instrument for registration of all SCs and pipes  - optional (not installed in this project): tensiometers for vacuum control  Material per campaign:  - cooling boxes with plastic bottles |
| initial costs and time | low  - time for side tunnels during first installation | low | high  - costs for purchase of material  - installation: 4 days per field with 4 people |
| returning time per strip  (without transport) | per device:  - 5 min material preparation  - 25 min field work (location with GPS, excavation)  - 30 min sample preparation and laboratory  **🡪 1h / device / year**  **🡪 4h / strip / year**  **🡪 12h / field / year** | per 10 samples (0-90 cm) in a strip:  - 15 min field work  - 105 min sample preparation (sieving, filtration) and laboratory  **🡪 2h / strip / campaign**  **🡪 4h / strip / year**  **🡪 12 h / field / year** | per 8 SCs in a strip:  - 5 min material preparation  - 30 min of field work  - 15 min of sample preparation (filtration)  **🡪 50 min / strip / campaign**  **🡪 10 h / strip / year**  🡪 **30 h / field / year** |
| laboratory equipment | - separation disks  - extraction solution  - analytical device | - soil sieves  - extraction solution  - analytical device | - filters  - ion chromatograph |
| dismantling costs | low | none | high  - all material incl. tubing system have to be removed (plastic and metal)  - use GPS points from installation |


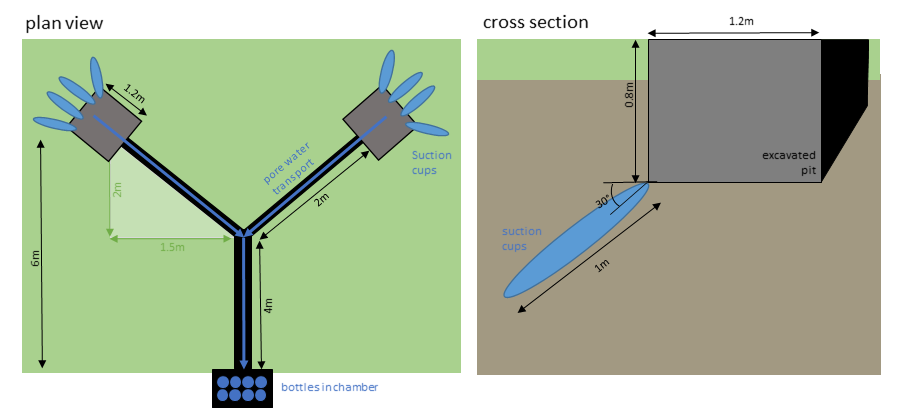
Figure 9: Details on suction cups installation per strip.


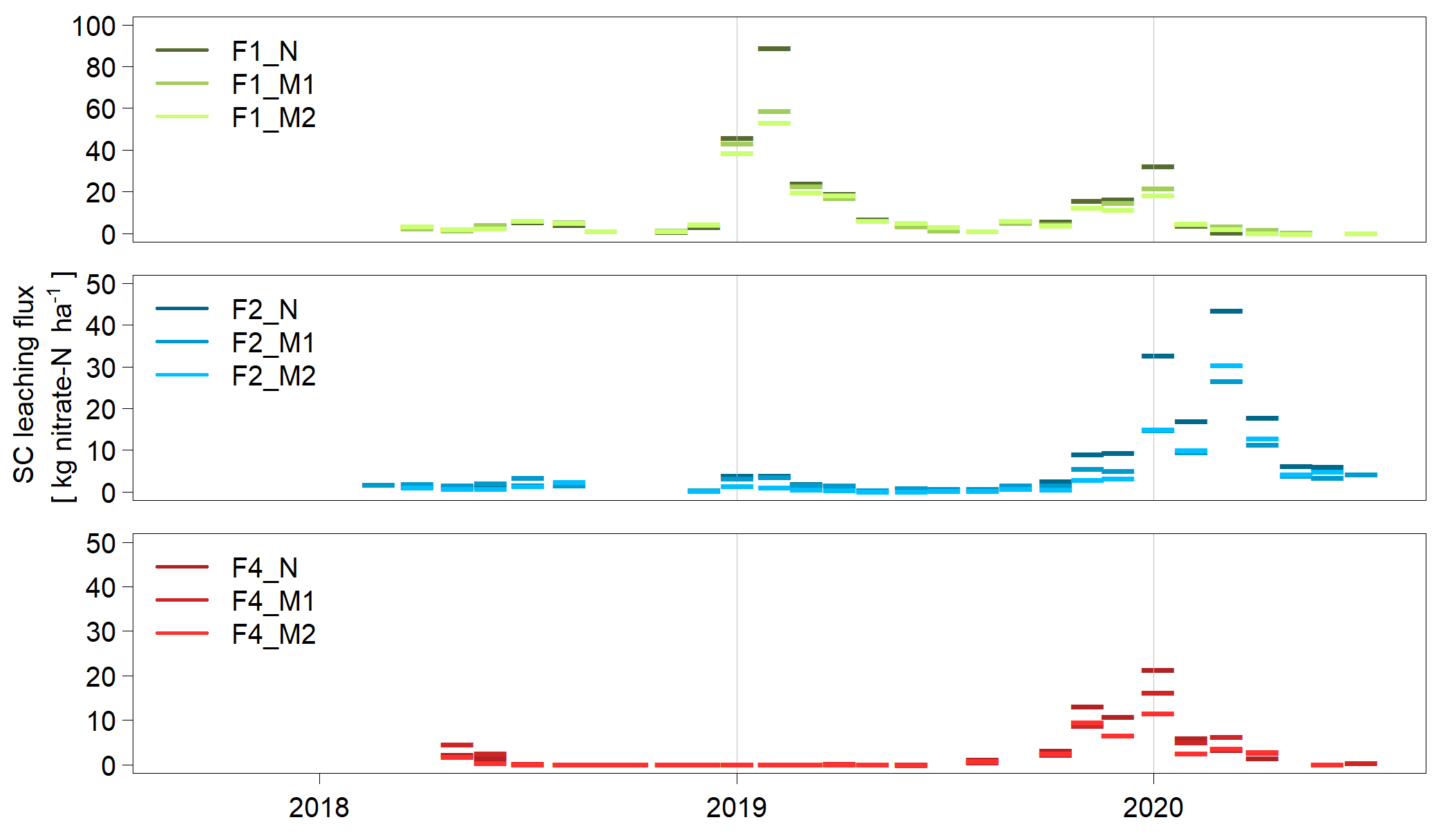


Figure 10: Monthly SC fluxes, calculated by multiplication of the mean SC concentration in that specific field and month with the cumulated water flux from the same period, derived with HYDRUS 1D.


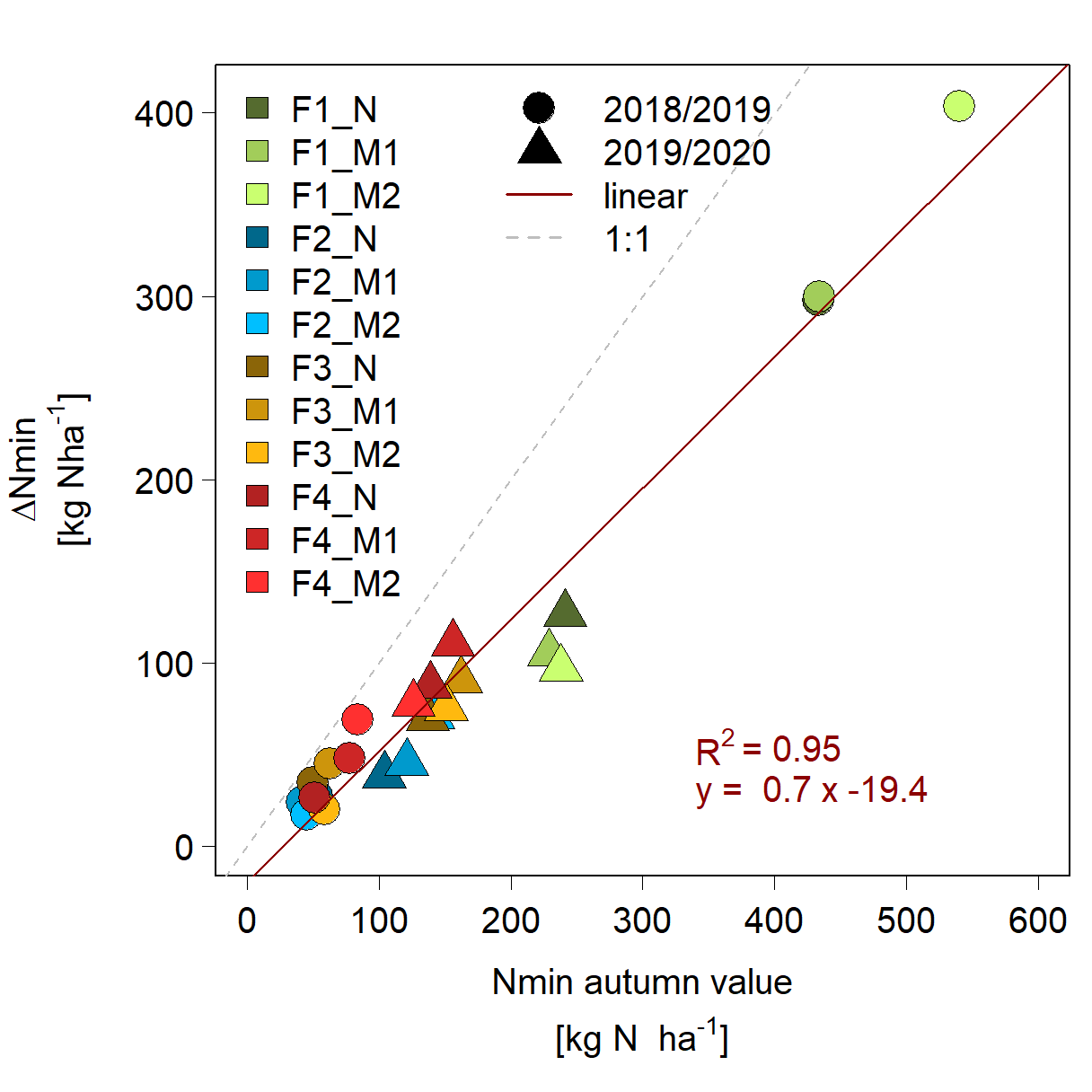


Figure 11: Analysis of the Nmin dataset. ∆Nmin refers to the difference between spring and autumn Nmin values.

Table 9: Detailed fertiliser applications with type and amount on all strips.

| **field** | **crop** | **date** | **fertiliser type** | **applied units**  **(kg N ha^-1^) *** | | |
| --- | --- | --- | --- | --- | --- | --- |
|  |  |  |  | **N** | **M1** | **M2** |
| H1 | grass-clover ley | August 2017 | nitrophos | 20 | | |
|  | grass-clover ley | September 2017 | cattle manure | 74 | | |
|  | maize | March 2018 | cattle dung | 75 | | |
|  | maize | June 2018 | urea 46% | 92 | | |
|  | wheat | February 2019 | cattle manure | 42 | 42 | 42 |
|  | wheat | April 2019 | urea 46% | 46 | 46 | 46 |
|  | wheat | May 2019 | ammonium nitrate 27 % | 54 | 54 | 0 |
|  | canola | February 2020 | B - ammonium nitrate 27 % | 133 | 81 | 0 |
|  | canola | March 2020 | B - ammonium nitrate 27 % | 0 | 52 | 0 |
| H2/3 | grass-clover ley | July 2017 | cattle manure | 53 | | |
|  | grass-clover ley | August.2017 | ammonium nitrate 27 % | 27 | | |
|  | grass-clover ley | February 2018 | cattle manure | 68 | | |
|  | grass-clover ley | March 2018 | cattle manure | 47 | | |
|  | grass-clover ley | May 2018 | cattle manure | 50 | | |
|  | grass-clover ley | June 2018 | cattle manure | 36 | | |
|  | grass-clover ley | July 2018 | cattle manure | 53 | | |
|  | grass-clover ley | August 2018 | cattle manure | 27 | | |
|  | grass-clover ley | February 2019 | cattle manure | 36 | | |
|  | maize | May 2019 | cattle manure | 29 | 0 | 0 |
|  | maize | May 2019 | di-ammonium phosphate | 27 | 27 | 27 |
|  | maize | June 2019 | urea 46 % | 83 | 83 | 37 |
|  | spelt | March 2020 | cattle manure | 18 | 18 | 18 |
|  | spelt | April 2020 | ammonium nitrate 27 % | 45 | 27 | 0 |
| H4 | spelt | March 2018 | cattle manure | 37 | | |
|  | spelt | April 2018 | Mg - ammonium nitrate | 29 | | |
|  | canola | August 2018 | digestate | 58 | | |
|  | canola | September 2018 | cattle manure | 31 | | |
|  | canola | February 2019 | B - ammonium nitrate 27 %  Ammonium Sulfate *** | 70  - | 70  - | -  100** |
|  | canola | March 2019 | B - ammonium nitrate 27 % | 70 | 39 | 0 |
|  | barley | September 2019 | Cattle manure | 44 | 44 | 44 |
|  | barley | March 2020 | Mg ammonium nitrate  Ammonium Sulfate *** | 36  - | 36  - | -  90** |
|  | barley | April 2020 | Urea 46 % | 69 | 46 | 0 |

Table 10: Flow-weighted nitrate concentrations for the periods 2018/19 and 2019/20.

|  | F1_N | F1_M1 | F1_M2 | F2_N | F2_M1 | F2_M2 | F4_N | F4_M1 | F4_M2 |
| --- | --- | --- | --- | --- | --- | --- | --- | --- | --- |
| 2018/19 | 256.5 | 211.2 | 199.8 | 17.5 | 17.5 | 6.1 | 1.0 | 1.1 | 1.0 |
| 2019/20 | 63.0 | 54.0 | 46.6 | 109.2 | 69.0 | 66.6 | 47.2 | 38.3 | 31.7 |
